# Designing a multi-epitope based vaccine to combat Kaposi Sarcoma utilizing immunoinformatics approach

**DOI:** 10.1038/s41598-019-39299-8

**Published:** 2019-02-21

**Authors:** Varun Chauhan, Tripti Rungta, Kapil Goyal, Mini P. Singh

**Affiliations:** Department of Virology, Post Graduate Institute of Medical Education and Research (PGIMER), Chandigarh, Punjab 160012 India

## Abstract

Kaposi’s sarcoma-associated herpesvirus (KSHV) responsible for causing Kaposi sarcoma (KS), an opportunistic angioproliferative neoplasm is emerging rapidly. Despite this there is no permanent cure for this disease. The present study was aimed to design a multi-epitope based vaccine targeting the major glycoproteins of KSHV which plays an important role in the virus entry. After the application of rigorous immunoinformatics analysis and several immune filters, the multi-epitope vaccine was constructed, consisting of CD4, CD8 and IFN-γ inducing epitopes. Several physiochemical characteristics, allergenicity and antigenicity of the multi-epitope vaccine were analyzed in order to ensure its safety and immunogenicity. Further, the binding affinity and stability of the vaccine with Toll like receptor -9 (TLR-9) was analyzed by molecular docking and dynamics simulation studies. In addition, an *in silico* cloning was performed to ensure the expression and translation efficiency of the vaccine, utilizing pET-28a (+) vector. Such T-cell-based immunotherapies which leverage this mechanism could prove their potential against cancer. Further, the authors propose to test the present findings in the lab settings to ensure the safety, immunogenicity and efficacy of the presented vaccine which may help in controlling KSHV infection.

## Introduction

KSHV, formally named as Human Herpes Virus-8 (HHV-8), is responsible for causing cancer - Kaposi’s sarcoma, commonly occurring in Acquired Immune Deficiency Syndrome (AIDS) patients, multicentric Castleman’s disease, primary effusion lymphoma and KSHV inflammatory cytokine syndrome^1^. The AIDS patients have 1,00,000 fold greater chances of acquiring KS than general population^2^. There is no specific antiviral therapy for HHV-8. Some retrospective studies suggest that ganciclovir or foscarnet, the commonly used anti-herpesvirus drugs may prevent the occurrence of KS upto some extent^3^. However, Boivin *et al*., reported that intravenous ganciclovir or foscarnet therapy did not affect the HHV-8 DNA load, thereby suggesting that these drugs do not directly affect growth of latently infected KS cells^4^. The recommended therapy for patients with aggressive disease includes liposomal anthracyclines or mono- or combination chemotherapies including doxorubicin or vinblastine, vincristine, bleomycin, dacarbazine and etoposide. The highly active antiretroviral therapies (HAART) are givento AIDS-associated KS patients. In advanced/nonresponsive KS, the supplementation of paclitaxel is given along with HAART regimen. In case of organ transplant patients, a switch to rapamycin has shown some regression in KS. Despite the implication of HHV-8 in KS pathogenesis, anti-herpetic drugs have no role in KS treatment^5^.

Vaccine is the most effective method to prevent viral diseases, but at present there is no report describing a vaccine against HHV-8. To combat tumors and viral infections, immune responses plays one of the most critical roles. The vaccines designed by conventional methods comprise of large proteins or whole organism thus incorporating unnecessary antigenic load and the chances of inducing allergenic response is also increased. These limitations can be overcome by using peptide based vaccines which are composed of short immunogenic peptide fragments that could elicit the highly targeted immune responses, thereby avoiding the chances of allergenic response. The advancements in the field of computational biology helps in designing an effective strategy for vaccine synthesis^6,7^. Several immunoinformatics tools have been developed for prediction of antigenic epitopes with potential translational implications^8^. The immunoinformatics approach saves time duration, overcomes the hurdles of cost, and increases the chances of successful vaccine designing^9^. A multi-epitope vaccine comprising a series of peptides that could induce the activation of both humoral and adaptive immune response is an ideal strategy for prevention and treatment of viral or tumor infections^10–12^. The present study was aimed to design a multi-epitope vaccine against HHV-8 comprising of Cytotoxic T cell lymphocytes (CTL), Helper T cell lymphocytes (HTL), Interferon-gamma (IFN-γ) inducing and B epitopes.

## Results

### Protein targets

**Figure 1:**
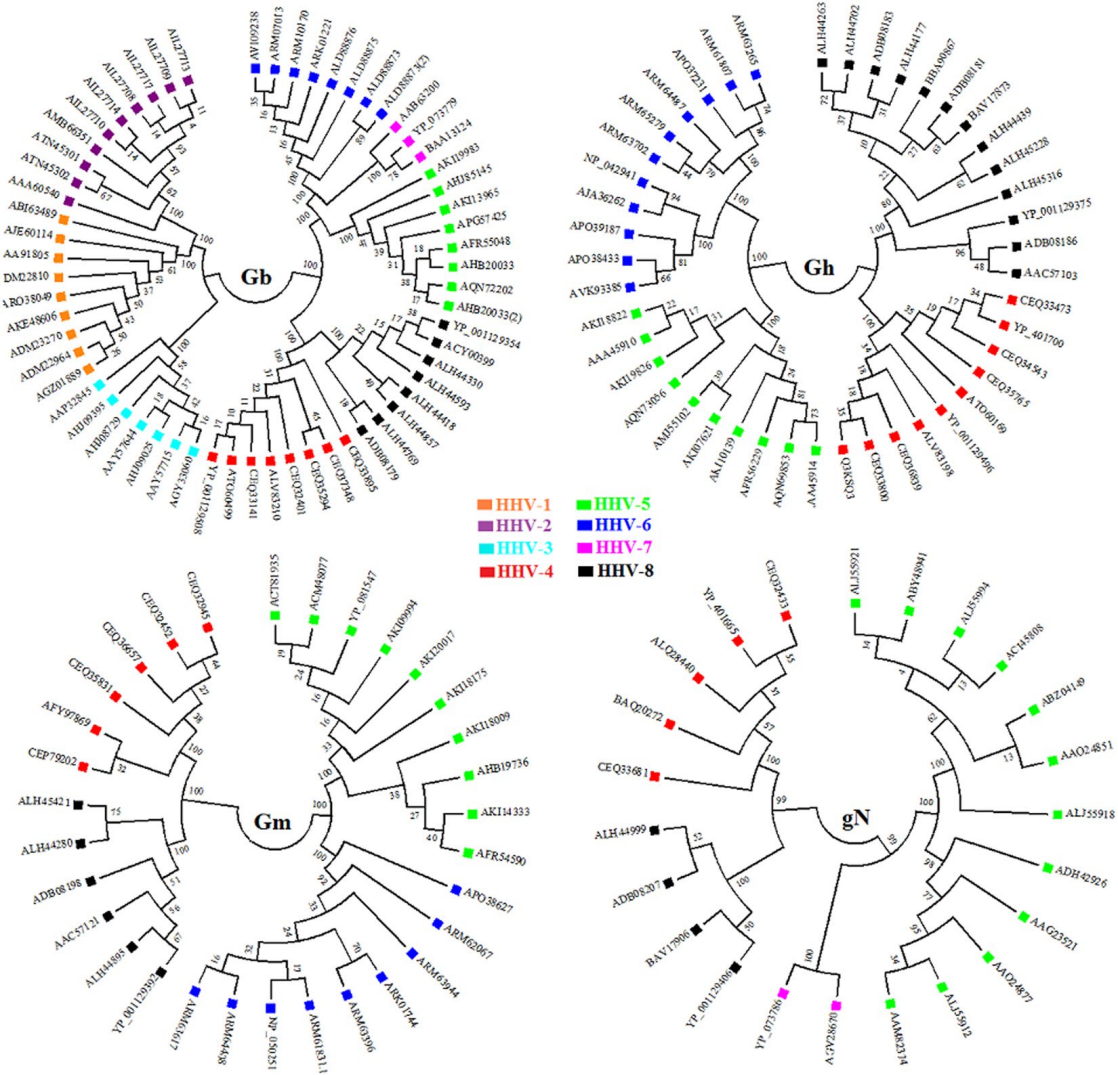
Phylogenetic analysis of target glycoproteins of HHV-8 using MEGA 7.0.14 software program. The accession numbers of the target glycoproteins from HHV-8 and from other members of the *Herpesviridae* family are indicated. At each branch the bootstrap values (1–100) are also indicated.

The NCBI database was utilized for the retrieval of amino acid sequences of the target glycoproteins. Further, the multiple sequence alignment of the sequences retrieved was carried out using Clustal W tool in Mega 7 without altering any parameter. The molecular analysis revealed about 41% similarity of HHV-8 Gb with HHV-4 (Epstein–Barr virus) at amino acid level, 33% with HHV-5 (Cytomegalovirus), 32% with HHV-6 and 7, 30% with HHV-2, 29% with HHV-1 and 27% with HHV-3(Varicella zoster virus). Similarly HHV-8 Gh showed 27%, 26% and 23% similarity with HHV-4, HHV-6 and HHV-5 respectively. The HHV-8 Gm showed 45%, 26% and 22% similarity with HHV-4, HHV-6 and HHV-5 respectively. The HHV-8 Gn showed 53%, 37% and 32% similarity with HHV-4, HHV-5 and HHV-7 respectively while the Gl of HHV-8 didn’t show significant similarity with any proteins with other members of the *Herpesviridae* family. Further the phylogenetic analysis was carried using UPGMA statistical method at 1000 bootstrap replications^13^ (Fig. 1). The phylogenetic analysis of gL of HHV-8 was not carried out as it didn’t show significant similarity with any of the proteins of other *Herpesviridae* family members. The accession numbers of all the target glycoproteins of HHV8 included in the present study is shown in Supplementary Table 1.

Further the target glycoproteins were also looked for identification of hits in human proteome. The database: Reference proteins (refseq_protein) using Blastp (protein-protein BLAST) was utilized for the same. The expected threshold value (E) was not altered and was kept 10. Other scoring parameters like matrix, gap costs and compositional adjustments kept were BLOSUM62, Existence:11 Extension: 1 and conditional compositional score matrix adjustment. None of the glycoproteins showed significant similarity with any of the proteins in human proteome (Supplementary Table 2). The Gl was found to be most antigenic followed by Gh, Gm, Gn and Gb with the antigenic scores of 0.646, 0.539, 0.538, 0.507 and 0.444 respectively, as predicted by VaxiJen 2.0 server. Other physicochemical characteristics like molecular weight, theoretical pI, half-life in human reticulocytes and the secondary structural properties of the target proteins were also predicted (Supplementary Table 3, Supplementary Fig. 1).

### 3D models preparation and validation

The 3D models retrieved using Phyre 2, Raptor X and I Tasser homology modeling tools were subjected to Ramachandran plot analysis. The details of the templates used by different homology modeling tools and the quality of the models retrieved were analyzed. The models retrieved from Raptor-X were of better quality and showed good Ramachandran plots as compared to those retrieved from other homology modeling tools like I-Tasser and Phyre-2 (Supplementary Table 4). In addition the detailed alignment statistics of the finalized models of the target proteins were also noted (Supplementary Table 5). Further the models were subjected to ModRefiner server for refinement. The details of the finalized structures and their Ramachandran plot results are shown in Table 1. As depicted in Table 1, the models generated by Raptor X server showed the best results and were considered for further analysis.

**Table 1:** Structural details of the models of the target proteins finalized.

Protein	Tool utilized for modeling	Template	Ramachandran Plot		
			Favored Region	Allowed Region	Disallowed Region
Gb	Raptor X	3fvcA	93.5%	5.5%	1.1%
Gh	Raptor X	5w0kA	94.6%	4.1%	1.2%
Gl	Raptor X	5w0kB	97%	3%	0%
Gm	Raptor X	1occA	95.3%	3.8%	0.9%
Gn	Raptor X	5x0 mA	96.3%	3.7%	0%

The models generated by Raptor-X were further subjected for refinement and finally the Ramachandran plot analysis of the finalized models were done.

### T cell epitopes prediction

**Figure 2:**
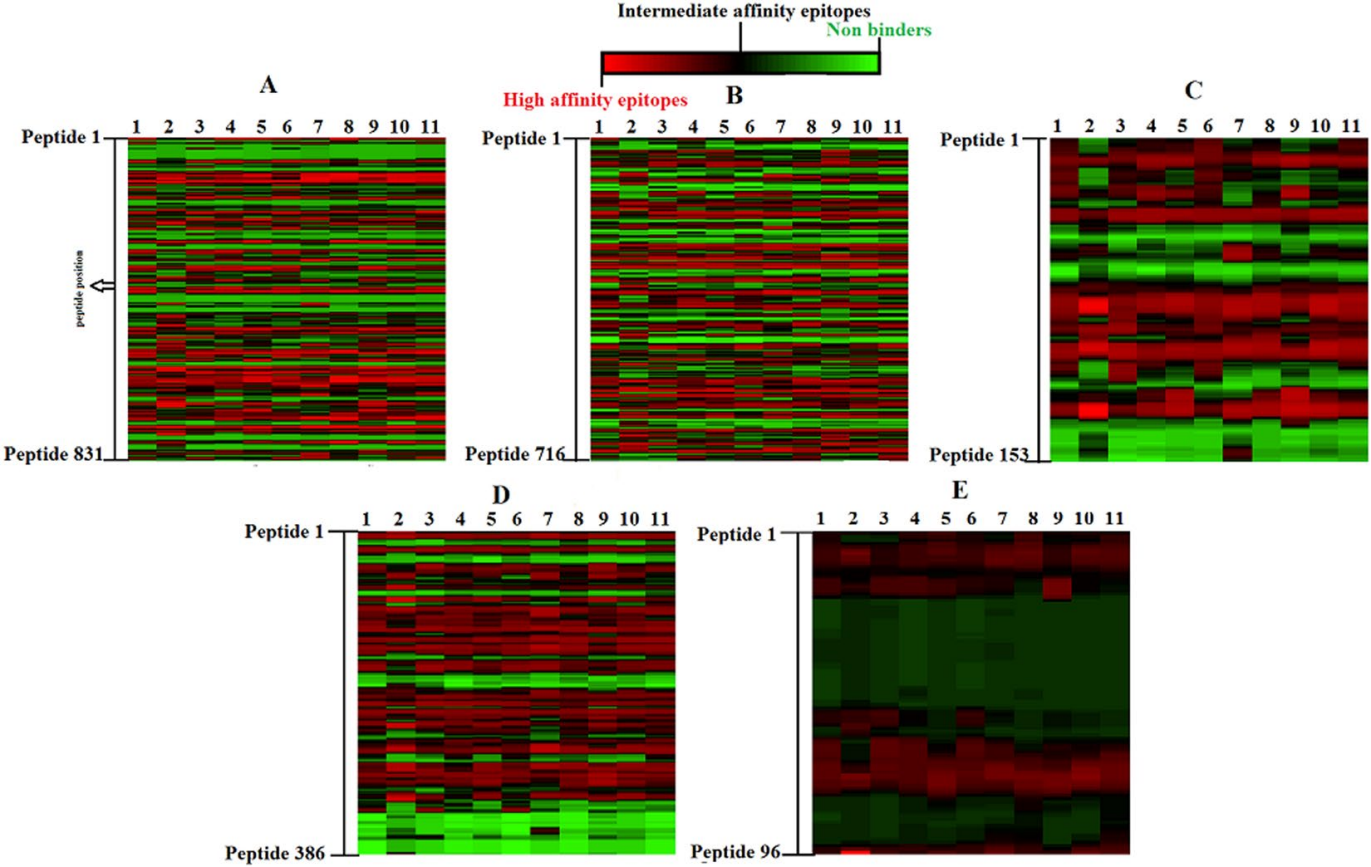
Heat maps depicting the frequency of strong, intermediate and low binding affinities of the epitopes towards different HLA alleles. (**A–E**) are the heat maps of Gb, Gh, Gl, Gm and Gn respectively. On the y axis are the epitopes and on x axis, the columns 1, 2, 3, 4, 5, 6, 7, 8, 9, 10 and 11 represent the following HLA-DRB1 alleles- *01:01, *03:01, *04:01, *07:01, *08:03, *10:01, *11:01, *12:01, *13:02, *14:01and *15:01 respectively.

**Figure 3:**
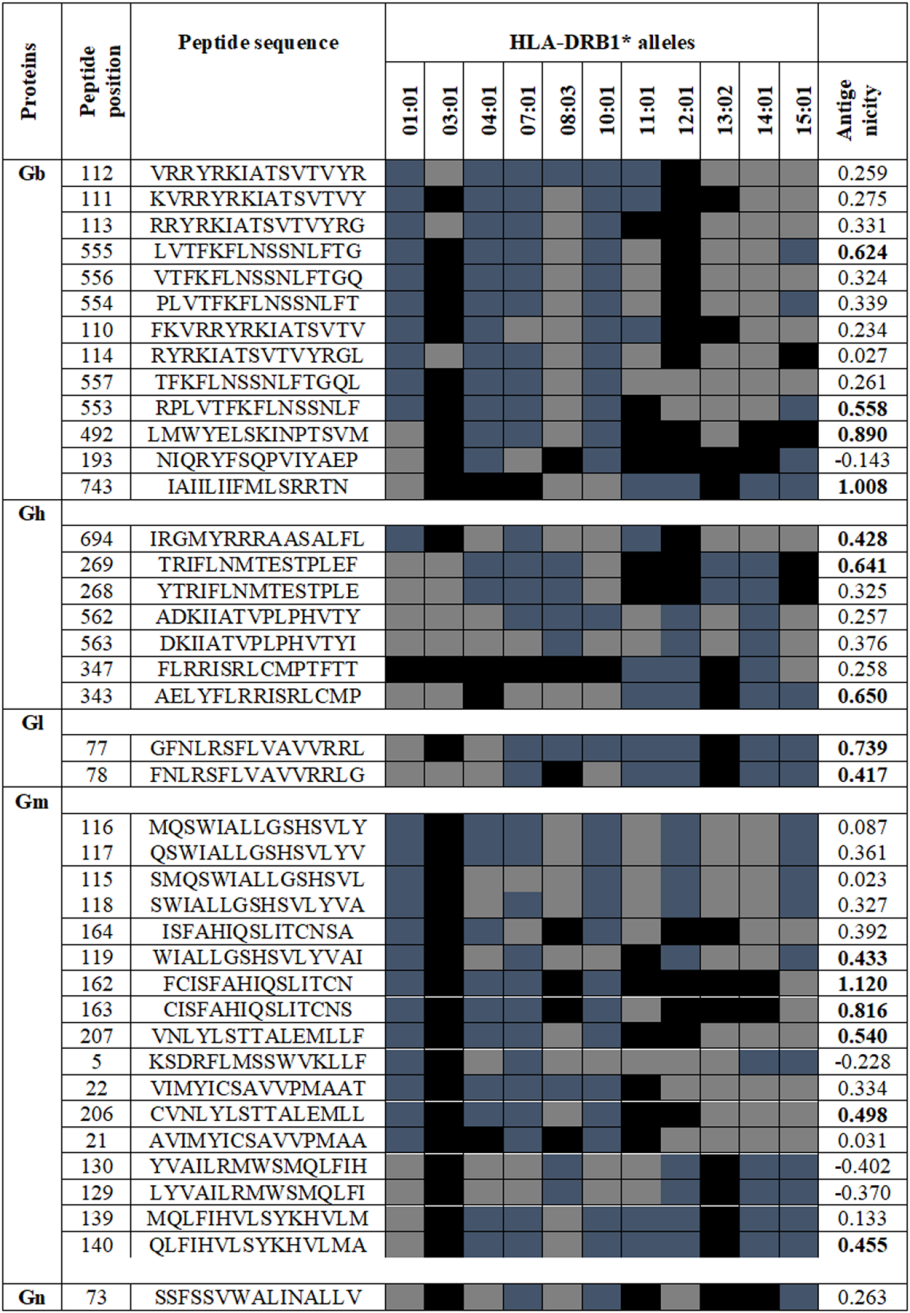
Promiscuous HTL epitopes. The boxes colored with blue, light grey and black shows the strong, intermediate and non-binding affinities towards the respective HLA alleles. The epitopes listed in the table showed 100% conservancy (as predicted by IEDB conservancy analysis tool) among the protein sequences included in the present study. The antigenicity of the epitopes was predicted using VaxiJen v2.0 tool and the values depicted in bold were predicted to be antigenic. The threshold kept for antigenicity prediction was 0.4.

The NetCTL 1.2 and IEDB consensus methods were utilized for CTL epitopes prediction and the HTL epitopes were predicted using Net MHC II pan 3.2 server and IEDB consensus method respectively. Longer the sequence of the target glycoproteins, more were the number of HTL epitopes predicted (Fig. 2). Further the epitopes which were predicted to have the strong binding affinities were subjected to various immune filters in order to screen out the best possible epitopes. The criteria designed for screening out the best possible epitopes was that it should be 100% conserved among the protein sequences included in the study, should be immunogenic, should not overlap with the glycosylation sites predicted in the respective proteins, should be non-allergic, should be promiscuous and should not overlap with any human proteins. Among the promiscuous CTL epitopes predicted, the Gb and Gm were predicted to have 7 antigenic epitopes, Gh- 4 and Gm- 1 epitope respectively. Based on all these parameters, the study led to the identification of some promiscuous epitopes (Table 2) (Fig. 3) and overlapping epitopes (Supplementary Table 6).

**Table 2:** Promiscuous CTL epitopes.

Protein	Peptide Sequence (Position)	HLA Class I alleles* and supertypes**	Antigenicity
Gb	SASITGELF (69)	A1, B58, B62, B3501, B5801	−0.027
	KIATSVTVY (117)	A1, A3, A26, B58, B62, B5801	0.24
	EISHMDSTY (147)	A1, B26, B62, B3501	**0.97**
	HMDSTYQCF (150)	A1, A3, A24, B39, B58, B62	**1.00**
	MIARSAEPY (227)	A1, A3, A26, B62, B3501	0.20
	TTHEDSFHF (329)	A1, A24, A26, B58, B62	**0.55**
	LTSDINTTL (363)	A1, A2, B39, B58	**0.49**
	HVPNGTVQY (382)	A1, A3, A26, B62, B3501	0.35
	RTNSKDVCY (543)	A1, A3, B58, B62, B5801	**1.72**
	ITRNETLVY (596)	A1, A3, A26, B58, B62	**0.61**
	TMFREYNYY (659)	A1, A3, A26, B58, B62	**0.58**
	ITATFTAPL (342)	A2, B7, B39, B58, B62	−0.19
Gh	LTFARNAKY (241)	A1, A3, A26, B58, B62	**0.75**
	FQMLVAHFL (314)	A2, B39, B44, A0201	−0.198
	RQYAELYFL (340)	A2, B27, B44, B62, A0201	**0.92**
	IAMVVEHMY (417)	A1, B58, B62, B3501, B5801	**0.54**
	MYTAYTYVY (424)	A1, A24, B62, B3501	0.22
	TAYTYVYTL (426)	A2, B39, B58, B5801	−0.28
	LMLDIHTVL (441)	A2, B8, B39, B62, A0201	0.36
	RTYLMFTSM (466)	A3, B7, B8, B58, B5801	**0.87**
	RAASALFLI (701)	A2, A24, B58, B5101, B5801	0.22
	YFLYRLFSI (720)	A2, A24, B8, B5101	0.29
	FLYRLFSIL (721)	A2, B8, B39, B62, B5101	−0.09
Gl	FAVLWTTLL (7)	A2, B7, B8, B39, B3501, B5101	−0.02
	TLLVTSHAY (13)	A1, A3, B62, B3501	**0.46**
Gm	MSSWVKLLF (11)	A1, A24, B58, B62, B3501, B5801	−0.11
	LLFVAVIMY (17)	A1, A3, B58, B62,	0.18
	AVVPMAATY (29)	A1, A3, B3501, B58, B62	**0.44**
	IVDGIAIVY (86)	A1, A3, B58, B62, B3501	**0.44**
	SMQSWIALL (115)	A2, B39, B62, A0201	−0.09
	RMWSMQLFI (135)	A2, B27, B58, A0201, B5801	−0.04
	SMQLFIHVL (138)	A2, B8, B39, B62, A0201	−0.63
	QLFIHVLSY (140)	A1, A3, A26, B62	−0.27
	HVLMAAFVY (150)	A1, A3, B58, B62, B3501	0.24
	NLYLSTTAL (208)	A2, B7, B8, B39, B62, A0201	**0.78**
	YLSTTALEM (210)	A1, A2, A62, A0201	**1.11**
	MMAVGNSFY (226)	A1, A3, A26, B62, B3501	**0.48**
	FLALTVVWY (248)	A1, A3, A26, B62	**1.17**
	LALTVVWYI (249)	A2, A24, B58, A0201, B5101, B5801	**0.69**
Gn	SVWALINAL (77)	A2, A26, B7, B62,	0.07

The epitopes listed in the table showed 100% conservancy (as predicted by IEDB conservancy analysis tool) among the protein sequences included in the present study. The antigenicity of the epitopes was predicted using VaxiJen v2.0 tool and the values depicted in bold were predicted to be antigenic. The threshold kept for antigenicity prediction was 0.4.

### B cell epitopes prediction

The BCpred 2.0 server was utilized for the prediction of linear/continuous B cell epitopes. Just like T cell epitopes, the screened out B cell epitopes were 100% conserved among the protein sequences included in the study. In addition to the parameters designed for screening the T cell epitopes, one additional parameter was added for screening out the B cell epitopes i.e. they should be located on the surface of the protein. Based on all these parameters, a total of 18 epitopes (gB-8, gH-5, gL-1, gM-3 and gN-1) were predicted for all the target proteins (Table 3). Further, the conformational/discontinuous epitopes were predicted using ElliPro server. A total 16 conformational B-cell epitopes (Gb-4, Gh-4, Gl-2, Gm-4 and Gn-2) were predicted, with the threshold 0.7 and the maximum distance 6 (Supplementary Fig. 2). In addition, the IFNepitope server was utilized for IFN-γ inducing epitopes prediction (Supplementary Table 7).

**Table 3:** The predicted Linear B cell epitopes utilizing BCpred 2.0 server.

Glycoproteins	B cell epitopes (position)
Gb	QTSSSPTPPGSSSKAPTKPG (32)
	NLEQTCPDTKDKYHQEGILL (80)
	TESAITNKYELPRPVPLYEI (129)
	KVNVNGVENTFTDRDDVNTT (162)
	SQPVIYAEPGWFPGIYRVRT (199)
	TNNQVETCKDTCEHYFITRN (580)
	IYPDVDRRAPPSGGAPTREE (767)
	HQLQQEERQKADDLKKSTPS (795)
Gh	PTTATTITRSATQLINGRTN (27)
	FARDTPEYRVFYPMNVMAVK (161)
	MVVEHMYTAYTYVYTLGDTE (419)
	DIHTVLTDSCPPKDSGVSEK (444)
	QIDRHIPIVYNISTPRRGCP (616)
Gl	AKLRSKTGDITVETCVNGFN (60)
Gm	VIRVIRSDWGLCTPSAAYMP (320)
	DRTPTVHQKPPPLPAKTRAR (348)
	ISTPAPRTQYQSDHESDSEI (373)
Gn	SARPSPGPTSVTTPGFYDVA (43)

### Multi-epitope vaccine- construction, structural properties and refinement

**Figure 4:**
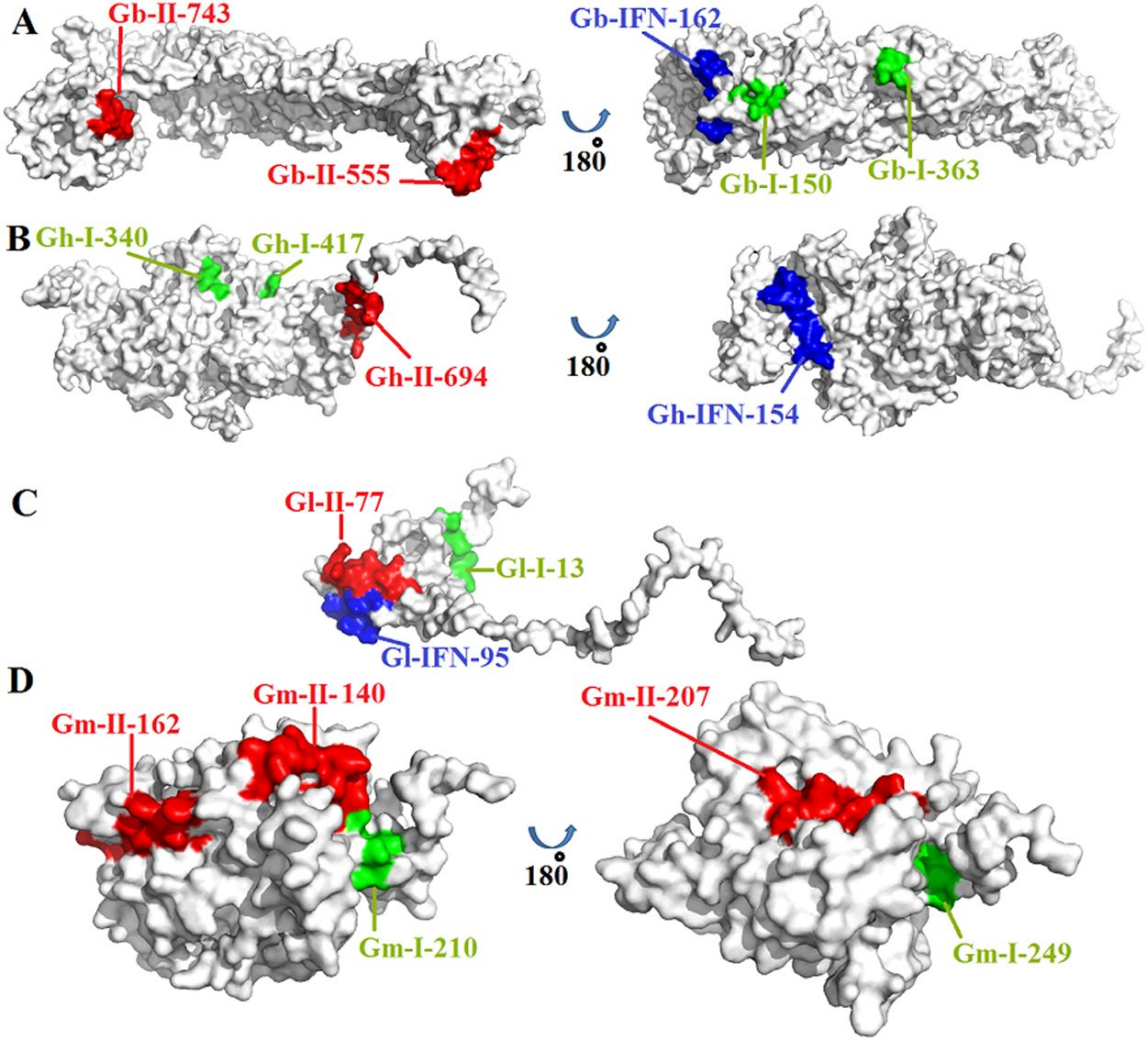
The 3D models of the glycoproteins showing the surface location of the epitopes included in the multi-epitope vaccine construct. (**A**) Glycoprotein B; (**B**) Glycoprotein H; (**C**) Glycoprotein L, (**D**) Glycoprotein M. The epitopes shown in red, green and blue colours are the HTL, CTL and IFN-γ epitopes.

**Figure 5:**
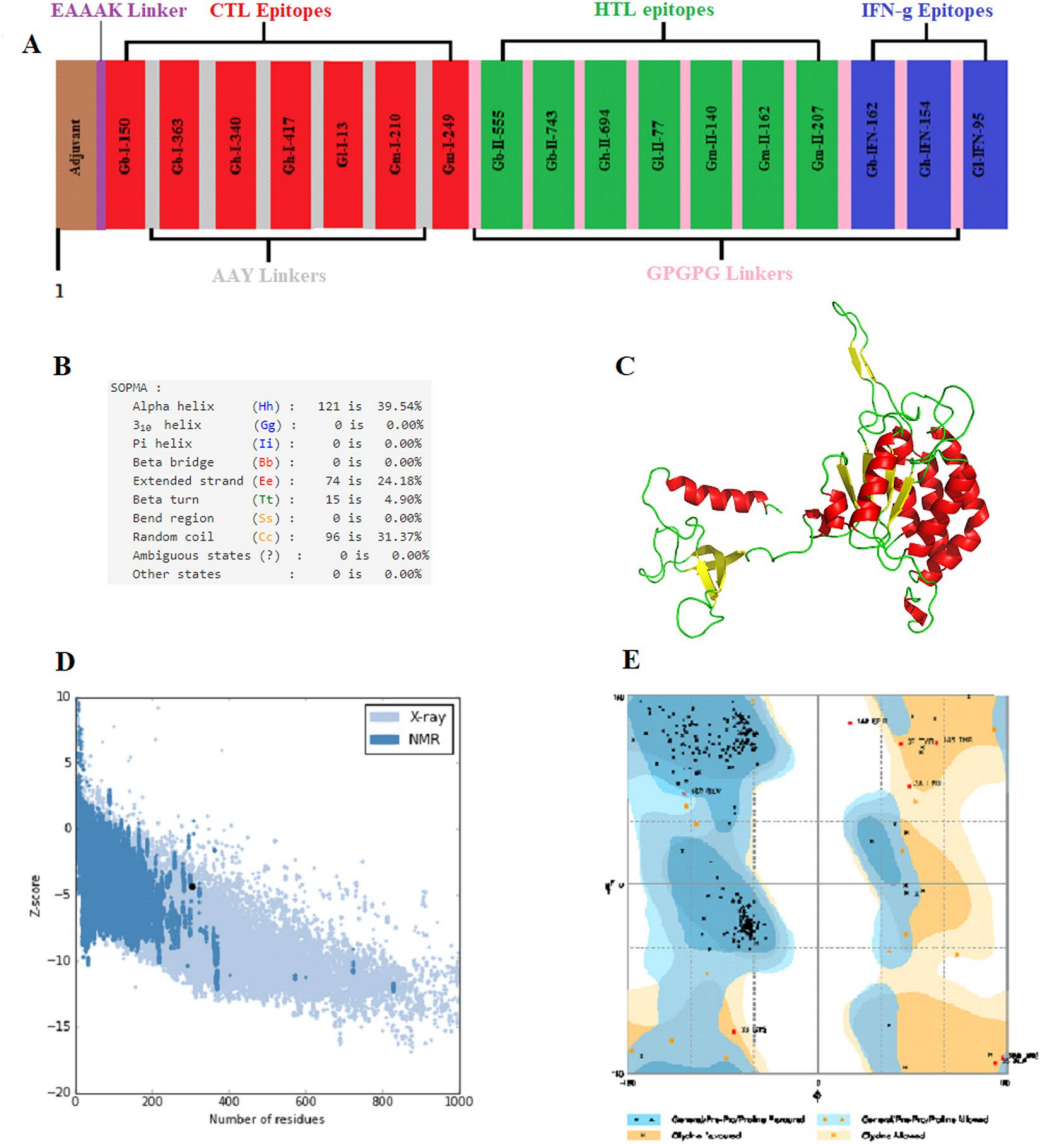
(**A**) Schematic view of final vaccine construct. The CTL HTL and IFN-γ epitopes included in the vaccine construct are shown in red, green and blue boxes. The CTL epitopes were linked to an adjuvant via EAAAK linker (purple) and the CTL epitopes were linked via AAY linker (Light grey). Similarly, the HTL and IFN-γ epitopes were linked via GPGPG linkers (light pink) respectively. (**B**) Secondary structure properties of the vaccine model. (**C**) 3D model of the final vaccine construct. Red, yellow and green colour represents the helical, sheet and loop region. (**D**) Validation of the structure with a Z score of −4.35 using PROSA. (**E**) Ramachandran plot analysis using Rampage server showing 92.8%, 4.3% and 3.0% in the favoured, allowed and outlier region.

The criteria designed for the epitopes to be included in the multi epitope vaccine construct was: (a) that they should be promiscuous, (b) should have overlapping CTL and HTL epitopes, (c) immunogenicity, (d) population coverage, (e) high affinity towards HLA alleles (predicted by docking analysis) and (f) should not overlap with any human gene (means it should not elicit autoimmunity). On the basis of these parameters, 7 CTL, 7 HTL and 3 IFN-γ inducing epitopes were selected. The population coverage of the individual CTL and HTL epitopes included in the vaccine are shown Supplementary table 8. Some epitopes showed even greater than 90% population coverage. The finalized epitopes were mapped on the surface of of their respective 3D models (Fig. 4). The final multi-epitope vaccine was constructed by linking the HTL and CTL epitopes via GPGPG and AAY linkers, respectively. The CpG-containing oligodeoxynucleotides were linked to the epitope chain via EAAAK linker (Fig. 5A). Now the final vaccine construct was ready comprising a length of 306 amino acids. The secondary structural properties of the vaccine showed that it was composed of 39.5% alpha helical regions, 24.18% extended strands, 4.90% beta turns and 31.37% random coil (Fig. 5B). The 3D model was constructed using Raptor-X online web server. The model retrieved was partitioned into two domains. A multi-template mode was selected for construction of the multi-epitope vaccine. The models with PDB IDs- 5kf6A, 4mpbA, 4k57A, 3u4jA and 2w8pA were utilized for the construction of domain 1 and the domain 2 was constructed using the template with PDB ID-5m41:A (Fig. 5C). Next the model was subjected to Galaxy Refine server for refinement. Among the refined models, the model 2 showed the best results in terms of the overall quality of the model prepared. Further the finalized model was subjected to ProSA-web to analyze the model quality. The results revealed the z score of −4.35 for the model constructed which was lying inside the scores range of the comparable sized native proteins (Fig. 5D). The overall quality of the finalized model of the multi-epitope vaccine construct was checked by Ramachandran plot analysis, the results revealed 92.8%, 4.3% and 3% residues lying in favored, allowed and outlier regions (Fig. 5E).

### Linear and conformational B cell epitopes and other physicochemical properties of the final vaccine construct

The amino acid sequence of the multi-epitope vaccine construct was predicted to be antigenic and non-allergenic with the immunogenicity score of 0.48 and allergenicity score of −0.93 as predicted by VaxiJen v2.0 tool and AlgPred server respectively. The linear/continuous and conformational/discontinuous B cell epitopes in the vaccine construct were predicted using BcPred 2.0 and Ellipro server without altering any prediction parameters. The servers predicted 9 linear/continuous and 7 conformational/discontinuous B cell epitopes (Table 4). The molecular weight, estimated half-life in human reticulocytes, theoretical pI, aliphatic index, instability index, and Grand average of hydropathicity (GRAVY) were predicted to be 32604.62 Da, 7.2 hours, 8.69, 86.18, 32.35 and 0.251 respectively.

**Table 4:** B cell epitopes (linear and conformational) in the final vaccine construct.

Sr. No.	Linear B cell epitopes (Position)	Scores	Conformational B cell epitopes	Scores
1	RTNGPGPGIRGMYRRRAASA (144)	1	YIGPGPGVNLY (105–115)	0.97
2	FTGGPGPGVNVNGVENTFTD (244)	1	TFKFLNSSNLFTGGPGPGVNVNGVENTFTDRDDGPGPGASKWSLFARDTPEYRGPGPGSQENLRLLWYLQRSL (234–298)	0.79
3	ARDTPEYRGPGPGSQENLRL (279)	1	ALTVVW (99–104) LSTT(116–119)	0.67
4	LTVVWYIGPGPGVNLYLSTT (100)	1	TCCATGACGTTCCTGACGTTE (1–21) AMVVEH (63–68) YAAYYLSTTALEMAAY (70–85)	0.64
5	FLGPGPGGFNLRSFLVAVVR (165)	1	Y(86) T(89) E(93)	0.57
6	KHVLMAGPGPGFCISFAHIQ (201)	0.99	Y (301) S(305) L(306)	0.52
7	TGACGTTCCTGACGTTEAAA (5)	0.99	YQCFAAYLTSDINTTLAAYRQY (31–52) NGPGPGI (140–152)	0.52
8	LITCNGPGPGLVTFKFLNSS (222)	0.96		
9	LEMLLFGPGPGIAIILIIFM (121)	0.90		

### TLR-9 modeling and molecular docking interaction with the final vaccine construct

**Figure 6:**
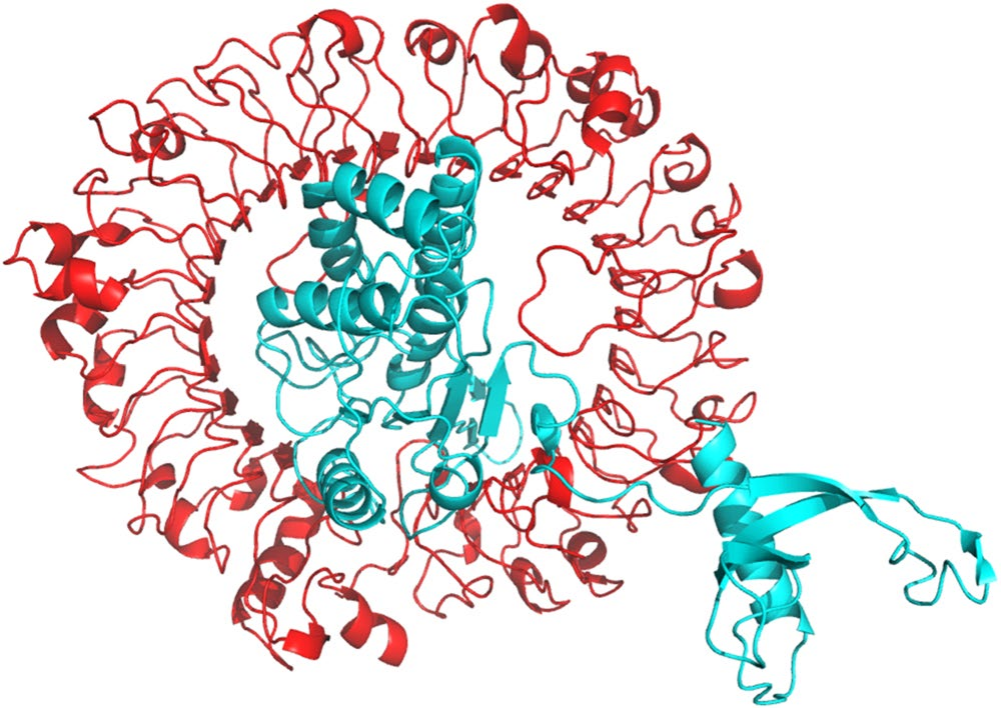
The interaction pattern of the proposed vaccine construct with TLR-9 predicted using Cluspro docking server. The vaccine and TLR-9 are shown in cyan and red color respectively.

**Figure 7:**
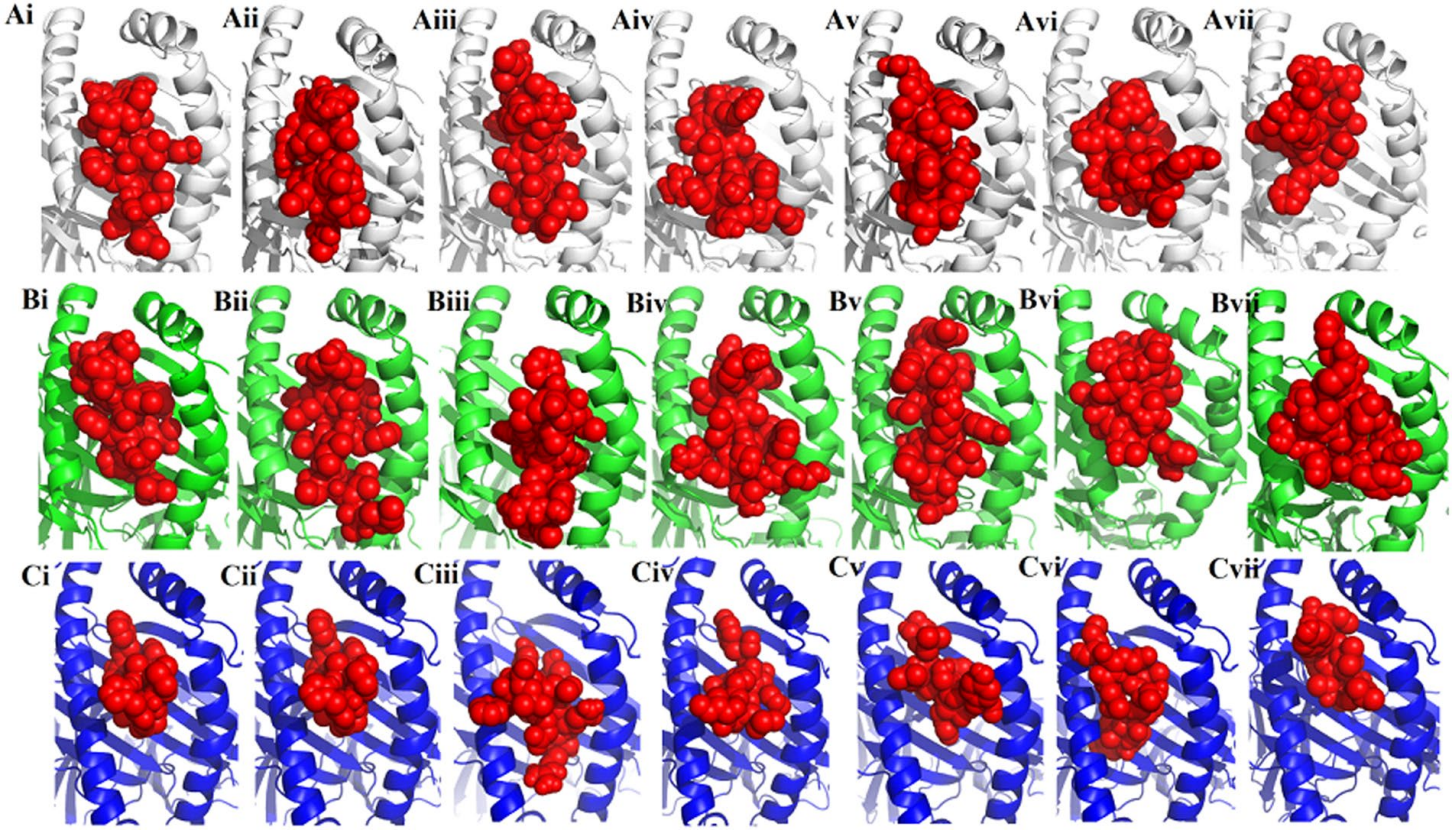
The binding patterns of the epitopes included in the final vaccine construct with HLA alleles. Ai-Avii and Bi-Bvii are the 7 HLA Class II T cell epitopes that are included in the final vaccine construct. The binding patterns of these epitopes with HLA Class II alleles: DRB1*01:01 (white), DRB1*15:01 (green) are shown. Similarly Ci-Cvii are the 7 HLA Class I T cell epitopes included in the vaccine construct, docked with HLA Class I allele - HLA-A*02:01 (blue). All the epitopes are shown as red spheres.

The 3D model of TLR 9 after homology modeling by Raptor X was of better quality as compared to that retrieved from I Tasser server. The model was subjected to ModRefiner server for refinement. Further in order to analyze the overall quality of the finalized model the Ramachandran plot was constructed using Rampage server. The numbers of residues in favoured region were 95.6%, 3.2% in allowed and 1.2% in outlier region (Supplementary Fig. 3). The interaction between the TLR-9 and the final vaccine construct was analyzed by molecular docking analysis using Cluspro. The Cluspro docking output gives multiple results, the top 10 generated models were elected for further analysis among which the result number 2 showed the best results in terms of docking interactions pattern between receptor and ligand, showing the lowest energy weighted score of −1881.2 kcal/mol (Fig. 6). The amino acids involved in the interaction between TLR-9 and the vaccine construct is shown in supplementary figure 4. In addition, the CTL and HTL epitopes were also docked individually with the commonly occurring alleles: HLA-A*02:01 (HLA Class I allele) and HLA-DRB1*01:01 and 15:01 (HLA Class II alleles) respectively to analyze their binding patterns (Fig. 7).

### Molecular dynamics

**Figure 8:**
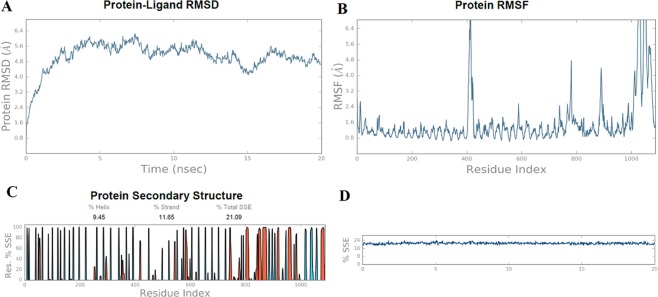
Molecular dynamics simulation results. (**A**) The RMSD plot of the docked multi-epitope vaccine (ligand) and TLR-9 (receptor) complex at 20 ns. The mild fluctuations indicates the stability of the vaccine and TLR-9 docked complex. (**B**) The RMSF plot of the docked complex. The high fluctuations indicate the flexibility in the docked complex. (**C,D**) Protein secondary structure elements analysis at the time of molecular dynamics simulation.

The molecular dynamics simulation was performed to investigate the physical movements of atoms and molecules of the final multi-epitope vaccine construct. During simulation its energy components, density, pressure, temperature and volume were evaluated and their stabilities were confirmed. The stability of the structure was analyzed via the root square deviation (RMSD) and the root mean square fluctuation (RMSF) of the protein backbone and all side chain residues respectively, of the multi-epitope vaccine construct for the time period of 20 ns. The RMSD of the complex of TLR-9 (receptor) and multi-epitope vaccine (ligand) was found to be 4.7 Å (Fig. 8A). Further the RMSF of amino acid side chains was noted to analyze the stability of the ligand-receptor interaction (Fig. 8B). The fluctuations in the RMSD plot were very mild thereby reflecting the uninterrupted interaction between ligand and receptor. In contrast, the fluctuations in the RMSF plot were very high indicating the flexibility in the regions between ligand-receptor complex. In RMSF plot, the peaks were very high (even greater than 6.4 Å) indicating the high degree of flexibility in the receptor-ligand complex. The protein secondary structure elements (SSE) were also monitored throughout the simulation (Fig. 8C,D).

### *In-silico* Cloning

**Figure 9:**
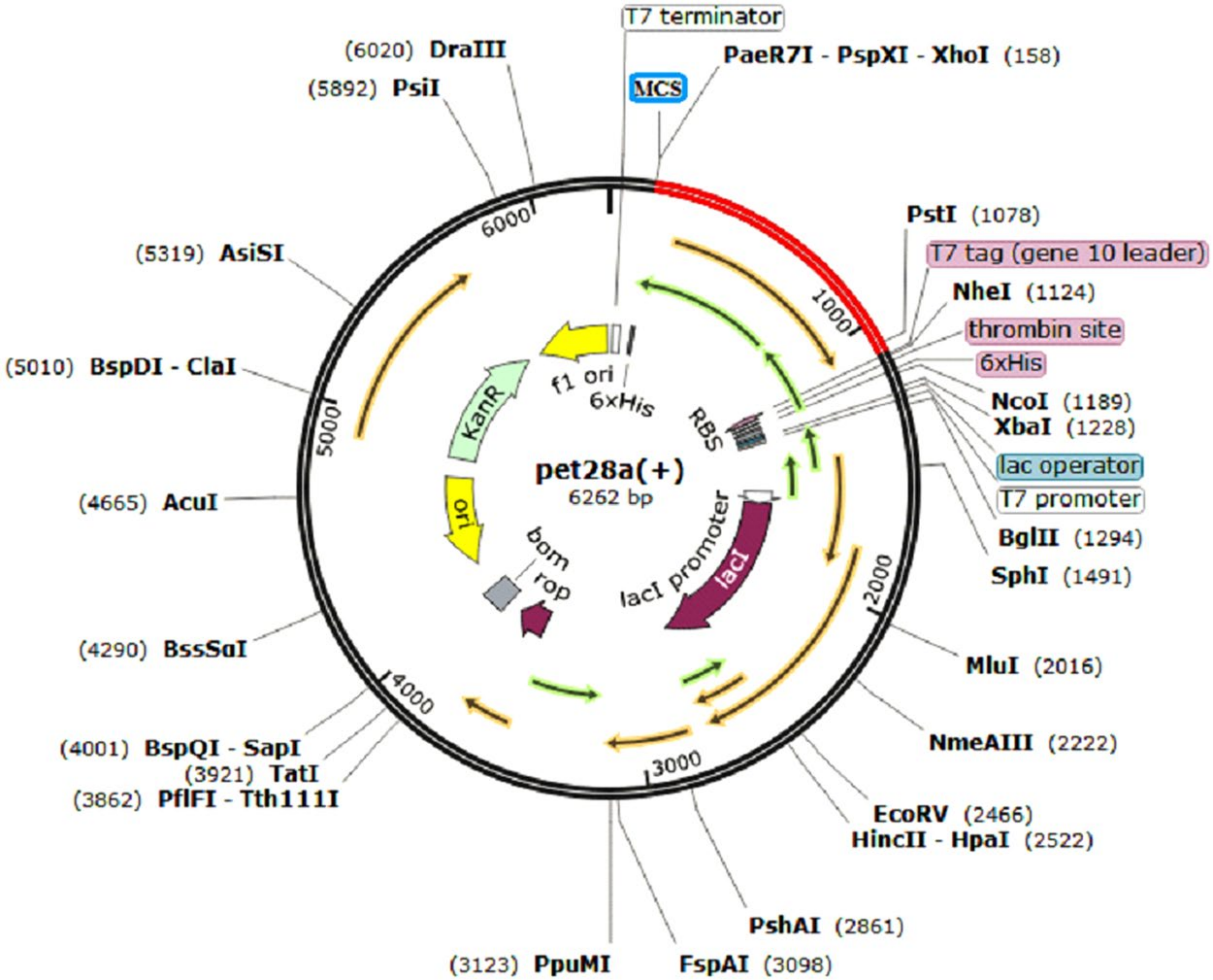
*In silico* restriction cloning. The red portion represents the multi-epitope vaccine insert into the pET28a(+) expression vector (black).

The cloning and expression efficiency of the multi-epitope vaccine construct in the expression vector was also analyzed by *in-silico* cloning. The Java Codon Adaptation Tool was used for optimizing the codon usage of vaccine construct in *E. coli* (strain K12). The optimized codon sequence was composed of 924 nucleotides. The Codon Adaptation Index (CAI) and the GC content of the optimized nucleotide sequence of the multi-epitope vaccine was 1.0 and 62.67% respectively, thus indicating the possibility of efficient expression of the vaccine in the host (*E. coli* -strain K12). Finally, the SnapGene tool was used for inserting the adapted codon sequences (multi-epitope vaccine sequence) in pET28a (+) vector for restriction cloning (Fig. 9).

## Discussion

Kaposi Sarcoma is an opportunistic angioproliferative neoplasmwhich presents most frequently as a cutaneous malignancy in patients who are immunocompromised, HIV patients (AIDS-related KS), and organ transplant recipients (iatrogenic KS)^14^. The increased occurrence of KS in immunosuppressed and organ transplant patients has focused the increased attention of pathologists. In fact, the incidence of KS among such patients is even 400–500 times higher than the general population^15^. There is no vaccine reported so far for this deadly disease. Thus keeping these points under consideration, the present study was aimed to design a multi-epitope vaccine for HHV-8 targeting its major glycoproteins, which facilitates the virus entry into the host.

The present study focused on designing a multi-epitope vaccine, as such vaccines have an edge over classical and single-epitope based vaccines due to the following unique properties: (a) it consists of multiple HLA epitopes that can be recognized by multiple TCRs; (b) it may consists of overlapping CTL, HTL and B-cell epitopes, thus has the capability to induce the activation of both cellular and humoral immune responses; (c) it consists of multiple epitopes from different target proteins/antigens of the target disease, thereby increasing the range of targeted virus; (d) in order to provide the long-lasting immune responses, the vaccine epitopes can be linked with adjuvant thereby enhancing its immunogenicity; and (e) the chances of adverse effects or pathological immune responses are reduced as it less likely contains such unwanted components^11,12,16–18^. Thus, a multi-epitope vaccine designed carefully using such a strategy could become a powerful tool to combat tumors and viral infections^19^.

The selection of appropriate antigenic epitopes of the target proteins to be included in the muti-epitope vaccine construct using *in silico* biological methods is extremely important^20^. The epitopes (T cell, B cell and IFN-γ epitopes) of the target glycoproteins identified in the present study were those which passed the several immune filters designed for screening out the epitopes. The filters applied were: the epitopes should be 100% conserved among the protein sequences, should be promiscuous, present on the surface of the target proteins, should not overlap with the glycosylation sites predicted in the respective proteins, should be immunogenic, should be non-allergic, should be promiscuous and should not overlap with any human proteins, thus reducing the chances of autoimmunity. Allergenicity is one of the major problems encountered during vaccine development. Thus allergenicity assessment is imperative at an early stage of vaccine designing. The epitopes screened out were first subjected to allergenicity assessment before designing of the final vaccine construct. In addition, the allergenicity of the final vaccine construct was also analyzed and it was observed to be non-allergen. Thus after a rigorous *in-silico* analysis, the final vaccine construct was designed.

**Figure 10:**
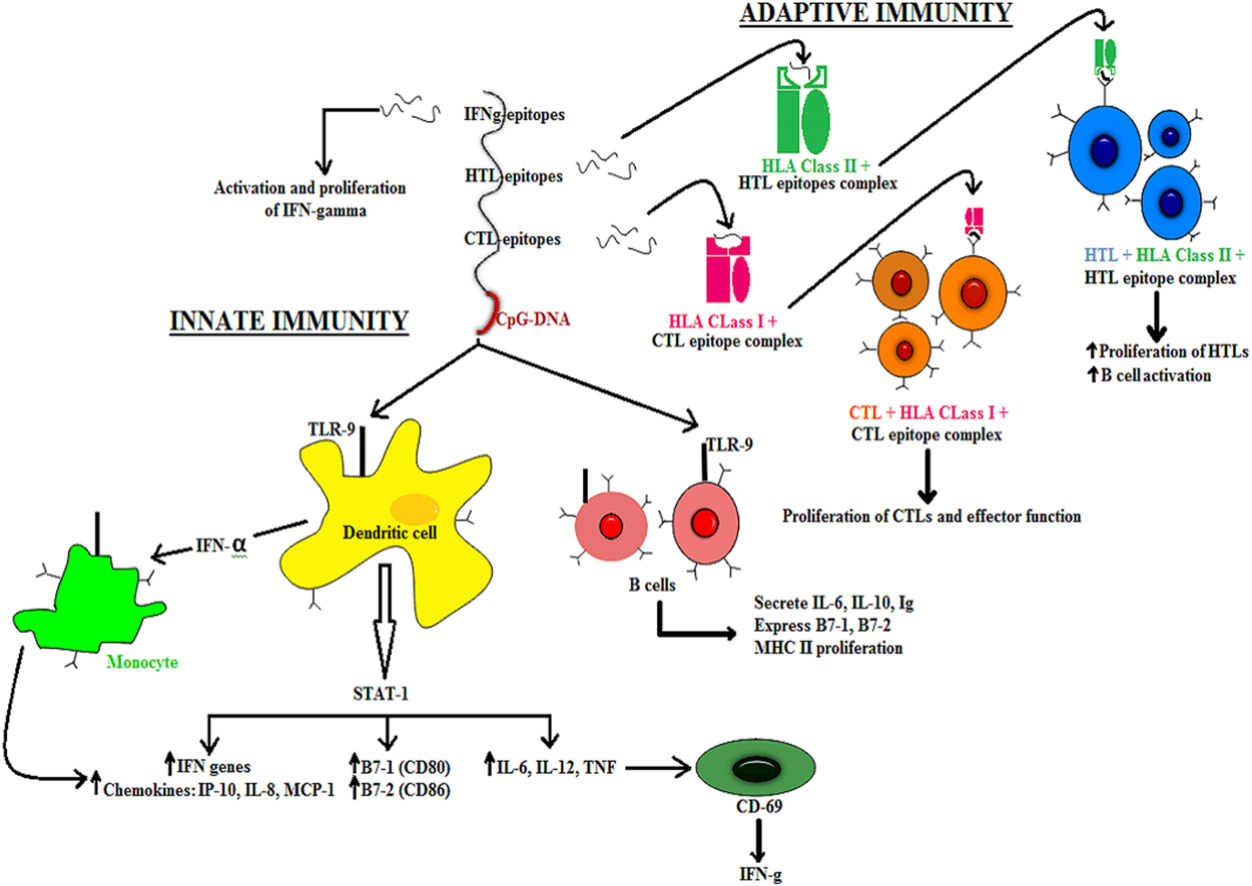
Proposed mechanism of action of the vaccine: The CpG (Oligonucleotides) ODNs will activate and interact with TLR-9 expressed on Dendritic cells, B cells, monocytes and macrophages which through complex reaction mechanisms will upregulate IP-10, IL-6, IL-8, IL-12, MCP-1, CD80, CD88 and IFN genes. Also, the other immune cells like NK cells, T cells, or other human monocytes will also be activated by CpG ODNs indirectly. In addition, the CTL and HTL epitopes will interact with HLA Class I and HLA Class II thereby forming epitope-HLA complex which in turn will interact with CTLs and HTLs, activating them and inducing their proliferation. The IFN-γ epitopes will activate the IFN genes. Thus the proposed vaccine has the ability to induce both adaptive and innate immune system.

As HHV-8 is a double stranded DNA virus, the Toll-Like Receptor-9 (TLR-9) expressed in the endoplasmic reticulum of Plasmacytoid dendritic cells should primarily recognize the structural components of the virus. There are several research articles which demonstrated the high levels of TLR-9 expression in HHV-8 infection^21–23^. The TLR-9 primarily express on the surface of plasmacytoid dendritic cells (pDCs) and B cells in humans. The TLR-9 is a well-known receptor for CpG^24^ and also the CpG motifs have been reported to trigger the cells which express TLR-9^25^. These CpG-ODNs have previously been reported as highly effective immunotherapeutics and superb adjuvants in vaccine against numerous infectious diseases like cancer, measles virus, hepatitis B virus, orthopox viruses, influenza virus, lymphocytic choriomeningitis virus, asthma, allergy, anthrax, leishmania etc^26^. The ODNs are divided into four types: D, K, C and P-type among which, the K type has been mainly focused as an adjuvant in human clinical trials against several infectious diseases and cancer^27^. Keeping these points under consideration, the CpG-Oligonucleotides (ODNs) belonging to K-type was implemented in the final vaccine construct which served as an adjuvant. The affinity and the interaction pattern analysis of the multi-epitope vaccine constructed with TLR-9 was analyzed by molecular docking analysis. Further the stabilization of the complex formed after TLR-9 and multiepitope vaccine interaction was analyzed by molecular dynamics approach. In designing such multi-epitope vaccine, its successful cloning and expression in an appropriate expression vector is again a very important step. Thus, the *in silico* cloning was performed in the present study to ensure the efficient expression and translation of the multi-epitope vaccine construct in an expression vector: pET-28a (+). The similar kind of strategy of designing a multi-epitope vaccine have been applied recently by several research groups against multiple myeloma, oncoprotein Her-2/neu, avian leukosis virus, Chikungunya, Hepatitis C virus, Dengue, Visceral leishmaniasis etc^12,28–33^. The proposed mechanism of action of the final vaccine construct was also predicted (Fig. 10). Since the vaccine consisted of CTL, HTL and IFN-γ epitopes, it could induce the activation of the respective immune cells in host, which may further lead to the activation of other immune cells through complex signaling.

## Conclusion

KSHV is an emerging infection in immunocompromised, HIV patients and organ transplant recipients. The statistical analysis indicates that the KHSV has grown rapidly among HIV infected patients. Despite this, there is no permanent cure/therapy or any vaccine reported for the disease. Many antivirals have been tested so far against KHSV, however none proved to be completely effective against the disease. In the present study, an attempt was made to design a mutiepitope vaccine against KSHV targeting its major glycoproteins. After applying numerous immune filters and a series of immunoinformatics tools, the final vaccine was constructed having 7 HTL, 7 CTL and 3 IFNγ inducing epitopes. Several B cell epitopes (both linear and conformational) were also identified in the multi-epitope vaccine construct. It was also predicted to be immunogenic and non-allergic. Thus the vaccine was designed in a way that it could induce the activation of innate, humoral and cell-mediated immune system. The adjuvant was also linked to the epitopes in order to enhance the immunogenicity of the vaccine; all were linked via suitable linkers for effective separation in the host. The mode of interaction between multi-epitope vaccine and TLR-9 was analyzed by molecular docking analysis and the stabilization of the ligand-receptor complex formed was checked using molecular dynamics simulation. The peptide-HLA complexes recognized by CTLs and HTLs cells are the cornerstone of cellular immunity for the elimination of infected or tumor cells. Thus, the molecular docking of the epitopes included in the final vaccine construct were also docked individually with HLA alleles for analyzing their affinities and binding patterns with different HLA alleles. Finally, the cloning, expression and translation efficiency of the multi-epitope vaccine construct was checked by *in silico* cloning assays. The authors further emphasize to validate the current findings in the experimental set up to ensure the efficacy and immunogenicity of the vaccine constructed.

## Methodology

### Retrieval of Amino acid sequences of the target proteins

The NCBI database was utilized for the retrieval of amino acid sequence in fasta format of all the target proteins. In addition, the amino acid sequences of the target proteins from other members of the *Herpesviridae* family were also taken into consideration in order to investigate the relatedness among them. Further the retrieved amino acid sequences were subjected to multiple sequence alignment tool- Clustal Omega to find out the conservancy among the target proteins. Further, the phylogenetic analysis was carried out to analyze the evolutionary divergence between them using MEGA 7.0.14 server.

### Analysis of the physicochemical and secondary structural properties of the target proteins

The physicochemical properties of the target glycoproteins were analyzed using the Expasy Protparam tool (http://web.expasy.org/protparam/). Further the antigenicity of each protein was determined using VaxiJen 2.0 server^34^. The threshold for predicting the probable antigen was set at 0.4. The four conformational states i.e. helix, sheet, turn and coils were predicted using SOPMA secondary structure analysis tool^35^. The parameters like number of conformational states, similarity threshold and window width were not altered while predicting the secondary structure of the target proteins.

### Homology modeling and validation

The widely used homology modeling tools - Phyre 2^36^, Raptor X^37^ and I Tasser (Iterative Threading ASSEmbly Refinement)^38^ were utilized for modeling the three dimensional (3D) structures of the target glycoproteins. The distortions in the structures retrieved after homology modeling were reduced using ModRefiner tool^39^ in order to refine the models. Next, the models were subjected to Ramachandran plot analysis using RAMPAGE server (http://mordred.bioc.cam.ac.uk/~rapper/rampage.php) to know the quality and reliability of the models prepared^40^. To validate the structure of the protein, the RAMPAGE server uses PROCHECK principle, the plots for Glycine and Proline residues are separated. The models thus showing the overall best results after all these analyses were selected for further use.

### T cell epitopes prediction

#### CTL epitopes

The 9-mer T cell epitopes recognized by the commonly occurring HLA Class I supertypes in human population i.e. A1, A2, A3, A24, A26, B7, B8, B27, B39, B44, B58 and B62 were predicted using the NetCTL.1.2 server^41^. The thresholds for different parameters like Transporter Associated with Antigen Processing (TAP) transport efficiency, proteasomal C-terminal cleavage, and epitope identification was set 0.05, 0.15 and 0.75 respectively, in NetCTL.1.2 server. In addition to this, the epitopes recognized by other HLA Class I alleles A-02:01, B-35:01 B-51:01 and A-58:01 were also identified utilizing Immune epitope database- consensus (IEDB) method^42^. The HLA Class I epitopes included in the study are expected to cover more than 90% of the worldwide population^43^. The peptides showing the consensus score of ≤2 were considered as strong binders and were selected for further analysis.

#### HTL epitopes

The epitopes of 15-mer length recognized by HLA Class II DRB1 alleles: 01:01, 03:01, 04:01, 07:01, 08:03, 10:01, 11:01, 12:01, 13:02, 14:01 and 15:01 were predicted using Net MHC II pan 3.2 server^44^ and IEDB consensus method^45^. The HLA Class II epitopes included in the study are expected to cover more than 95% of the worldwide population^43^. The peptides were categorized as strong, intermediate and non-binders based on the basis of percentile rank. The threshold for strong, weak and non-binding epitopes was kept 2%, 10% and greater than 10% respectively.

### Identification of promiscuous and overlapping T cell epitopes

The promiscuous epitopes are those that have the affinity towards multiple HLA Alleles. Such epitopes are of utmost importance in vaccine designing, as they are capable of generating effective immune response in host as they possess affinity towards multiple allelic forms of HLAs. Thus the screened out HLA Class I and Class II T cell epitopes were further assessed for promiscuousity analysis. The epitopes predicted to have high binding affinity towards multiple HLAs in the present study were screened out and were considered as promiscuous epitopes.

The overlapping epitopes have the capacity to induce the activation of cytotoxic T cells and helper T cells since such epitopes have integral sequences comprising of both CTL and HTL epitopes. Thus, in the present study, the HTL epitopes with high binding affinity which overlapped with the strong binding affinity CTL epitopes were listed out and merged as a single peptide fragment.

### Peptides immunogenicity prediction

The VaxiJen v2.0 tool (http://www.ddg-pharmfac.net/vaxijen/VaxiJen/VaxiJen.html) was utilized for analyzing the antigenicity of the predicted promiscuous epitopes. The antigenicity prediction threshold was kept 0.4.

### B cell epitopes identification

#### Linear B cell epitopes

The antigen (B cell epitopes) on interaction with the B cell lymphocytes stimulates them to differentiate into memory B cells and plasma cells. The linear/continuous B cell epitopes were identified using BCpred 2.0 server^46^. Further the predicted B cell epitopes were also assessed for beta turn prediction, surface accessibility, flexibility, antigenicity and hydrophilicity^47–51^.

#### Conformational B cell epitopes

The Ellipro server was utilized for prediction of conformational/discontinuous epitopes^52^. The minimum score and the maximum distance (Angstrom) was set to 0.7 and 6 Å respectively, as the prediction parameters while using Ellipro server.

#### Prediction of IFN-γ inducing epitopes

For both the arms of the immune system i.e. innate and adaptive, IFN-γ is the signature cytokine and plays an important role in eliciting anti-tumor, antiviral and immune regulatory activities. Thus identification of epitopes having the capacity to induce IFN-γ is important for designing an effective subunit vaccine. Thus, the IFN-γ inducing epitopes in the target proteins were predicted using IFNepitope server^53^.

#### Peptides conservation analysis and population coverage

The Immune Epitope Database (IEDB) Conservancy Analysis tool was utilized for the identification of the degree of conservancy of the predicted T and B cell epitopes in the protein sequences^53,54^. The epitopes showing the 100% conservancy were screened out for further analysis. Further the fraction of individuals predicted to respond to the screened out epitopes based on HLA genotypic frequencies was calculated using IEDB population coverage analysis^55^.

#### Characterization of the predicted epitopes

The physicochemical properties of the target glycoproteins were analyzed using the Expasy Protparam tool (http://web.expasy.org/protparam/). Further in order to reduce the chances of autoimmunity, the screened out epitopes were also subjected to BLASTP search against human proteome. The epitopes if found similar to any human protein were eliminated.

### Peptides and HLAs interaction pattern analysis

#### Modeling of peptides and HLA alleles

The online available PEPFOLD 3 server was employed for generating the 3D structures/pdb files of the epitopes^56^. The X-ray crystallographic structures of the three commonly occurring HLA alleles in human population i.e. HLA-DRB1*01:01 and 15:01 (HLA Class II alleles) and HLA-A*02:01(HLA Class I allele) were retrieved from Protein Data Bank (PDB) bearing PDB ID- 2g9h, 1BX2 and 1QEW respectively. Further the previously bound ligands to these alleles were removed and the energy minimization for the receptors (HLA alleles) was carried out.

#### Molecular Docking analysis

Molecular docking analysis is an important tool for understanding the protein-protein interaction patterns. In order to investigate the interaction patterns of the HLA alleles with the screened out epitopes, ClusPro (v.2), a rigid PIPER protein-protein docking web server based on the Fast Fourier Transform (FFT) was employed. The server performs rigid docking by sampling billions of conformations, pairwise root-mean-square deviation (RMSD) of the complex, and energy minimization. The server calculates the binding energy scores of the docked complex based on attributes such as Deocys as reference states (DARS), electrostatics, desolvation contribution and shape complementarity. The docking analysis was carried out under hydrophobic environment and the optimal model showing the binding of the epitopes in the peptide binding groove of HLA’s and also depending on the size of the cluster. Finally the docked complex was visualized using PYMOL version 1.7.4.4. (Schrodinger) in order to analyze the interaction pattern of the peptides with HLA alleles.

#### Toll Like Receptor 9 (TLR-9)- Homology Modeling and its structure validation

The amino acid sequence of TLR-9 was retrieved from UniProt with ID- Q9NR96. The tertiary structure was retrieved using the online homology modeling tools I-tasser and Raptor-X. The I-tasser server employs a fragment-based method for modeling and combines the methods of threading, comparative modeling, ab initio modeling and structural refinement. The server builds the 3D model of the protein based on the hierarchical approach. The models are generated based on threading alignments from PDB library in which the fragments from different templates structures are excised and then reassembled^57^. In CASP-7, 8 and 9 experiments, the I tasser server was ranked as the No. 1 for protein structure prediction^58^.

Another online web server, Raptor-X predicts the 3D models, disordered regions as well as solvent accessibility regions of the target proteins. The models predicted by this server is indicated by the several confidence scores, the absolute global quality of the model is predicted by global distance test (GDT) and un-normalized GDT (uGDT), and P-value and RMSD for the absolute local quality and relative global quality of each residue in the model respectively. Finally the refinement, quality and reliability of the model prepared were done as described in above section (homology modeling and validation). The model thus showing the overall best results was selected for further use.

#### Designing of final multi-epitope vaccine sequence construct

The CTL, HTL and IFN-γ inducing epitopes were included in the multi-epitope vaccine sequence. The epitopes selected for final vaccine construct were predicted to be highly promiscuous, overlapping, immunogenic and non-allergic. The AAY and GPGPG linkers were used for linking the CTL and HLT epitopes together respectively. In addition, the CpG-containing oligodeoxynucleotides was also included in the vaccine construct which act as adjuvant to improve the immunogenicity of the vaccine. It was linked to the multi-epitope sequence via EAAAK linker.

#### Prediction of antigenicity, allergenicity and various physicochemical properties

The antigenicity of the final vaccine construct was analyzed by VaxiJen v2.0 tool. The threshold for antigenicity prediction was kept 0.4. The allergenicity of the vaccine was evaluated using AlgPred server^59^. The ProtParam server was utilized for assessing various other physiochemical parameters of the vaccine like theoretical isoelectric point (pI), *in vitro* and *in vivo* half-life, amino acid composition, molecular weight, instability and aliphatic index, and grand average of hydropathicity (GRAVY).

#### Vaccine-structure modeling, refinement and validation

The SOPMA server was utilized for prediction of the secondary structural properties of the final vaccine construct. The 3D model of the vaccine was prepared using the homology modeling tool- Raptor-X, an online web server. Next the refinement of the model retrieved was carried out using Galaxy Refine server (http://galaxy.seoklab.org/). This server performs the repacking and molecular dynamics simulation to relax the structure, a CASP10 based refinement technique. The Galaxy Refine server is considered as one of the best performing algorithms to enhance the local structural quality, as per the CASP10 evaluation. The validation of the tertiary structure of vaccine was carried out using ProSA-web^60^. The server predicted the overall quality of the model which is indicated in the form of z-score. If the z-scores of the predicted model are outside the range of the characteristic for native proteins, it indicates the erroneous structure. Further the Ramachandran plot analysis of the predicted model of the vaccine was carried out to using RAMPAGE server to determine its overall quality.

#### Linear and Conformational epitopes prediction

The linear/continuous and conformational/discontinuous B cell epitopes in the final vaccine construct were predicted using BCpred 2.0 server and ElliPro^52^ respectively.

#### Molecular Docking of Toll Like Receptor-9 and multi-epitope vaccine

The interaction of the antigenic molecule/vaccine with the target immune cell receptor is necessary for the generation of an appropriate immune response. In order to analyze the interaction pattern of the multi-epitope vaccine with TLR-9, molecular docking analysis was carried out using Cluspro (Section 2.11.2). The results were visualized by Pymol (Schrodinger).

#### Molecular dynamics (MD) simulation of receptor (TLR-9) -ligand (multi-epitope vaccine) complex

The stability in the interaction of the protein-ligand structure was determined by MD simulations^33^. The High-performance molecular dynamics simulation was performed to understand the interaction pattern between the receptor (TLR-9) and the multi-epitope vaccine construct at the microscopic level by using Desmond (Schrodinger).

#### *In silico* cloning and optimization

The Java Codon Adaptation Tool (JCat) tool was utilized for reverse translation and codon optimization and expressing the multi-epitope vaccine in an appropriate expression vector. To express the final multi-epitope vaccine construct in *E. coli* - K12 strain, the codon optimization was carried out. The output of JCat includes codon adaptation index (CAI), the ideal CAI should be greater than 0.8 and scores upto 1.0. The ideal CAI score should be 1.0. The JCat tool also measures the GC content of the insert to ensure the high-level protein expression. Ideally the GC content percentage should range between 30–70%. If the value does not reside in this range then the translational and transcriptional efficiencies may be unfavorable. Finally, the SnapGene tool was utilized for inserting the optimized sequence of the multi-epitope vaccine into the pET-28a(+) vector.

## Supplementary information

Supplementary information
