# Supplementary material for: Designing a multi-epitope based vaccine to combat Kaposi Sarcoma utilizing immunoinformatics approach
Source: Sci Rep. 2019 Feb 21;9:2517. doi: 10.1038/s41598-019-39299-8 (PMC6385272; doi:10.1038/s41598-019-39299-8)
Supplement: Supplementary file 1 — Supplementary information [file 41598_2019_39299_MOESM1_ESM.docx]

**Designing a multi-epitope based vaccine to combat Kaposi Sarcoma utilizing immunoinformatics approach**

**Varun Chauhan^1^, Tripti Rungta^1^, Kapil Goyal^1^,Mini P Singh*^1^**

1- Department of Virology, Post Graduate Institute of Medical Education and Research (PGIMER), Chandigarh, India, Punjab-160012

| **Target glycoproteins** | **Accession numbers (retrieved from NCBI Database)** |
| --- | --- |
| Gb | YP_001129354, ACY00399, ALH44418, ALH44857, ALH44769, ALH44593, ALH44330, ADB08179, |
| Gh | YP_001129375, ADB08186, AAC57103, ALH44263, ALH44702, ALH44439, ADB08181, BBA90867, ALH45228, BAV17873, ADB08183, ALH45316, ALH44177, |
| Gl | YP_001129399, ALH44638, ALH44287, ADB08193, BAV17897, AHY94914, |
| Gm | YP_001129392, ALH44895, ALH44280, AAC57121, ALH45421, ADB08198 |
| Gn | YP_001129406, ADB08207, ALH44999 |

**Supplementary Table 1:** Accession numbers of the target glycoproteins of HHV8 retrieved from NCBI database included in the present study.

| **Target protein of HHV8** | **Query cover** | **E value** | **Identity** | **Human protein (Accession no)** |
| --- | --- | --- | --- | --- |
| Gb | 5% | 3.7 | 35% | sodium-independent sulfate anion transporter isoform X4 [Homo sapiens] (XP_024306478.1) |
| Gh | No similarity found | | | |
| Gl | 27% | 3.9 | 28% | sushi, von Willebrand factor type A, EGF and pentraxin domain-containing protein 1 precursor [Homo sapiens] (NP_699197.3) |
| Gm | No similarity found | | | |
| Gn | 38% | 0.50 | 38% | lamin tail domain-containing protein 2 isoform X5 [Homo sapiens] (XP_016872967.1) |

**Supplementary Table 2:** Blastp results of the target glycoproteins against the human proteome. The Gh and Gn didn’t showed any similarity with any of the proteins in human proteome. The Gb, Gl and Gn showed very low query coverage and identity, with the human proteome which was considered as insignificant. And also the epitopes predicted from these respective glycoproteins were also subjected individually to blast analysis against human proteome which again showed no significant identity with any of the human proteins.

| **Target proteins** | **Mol. Wt.** | **Theoretical pI** | **Half-life (in human reticulocytes, in vitro)** | **Antigenecity (Threshold 0.4)** |
| --- | --- | --- | --- | --- |
| Gb | 93984.52 | 8.31 | 30 hours | 0.444 |
| Gh | 81265.72 | 7.55 | 30 hours | 0.539 |
| Gl | 17998.80 | 10.14 | 30 hours | 0.646 |
| Gm | 45363.82 | 8.60 | 30 hours | 0.538 |
| Gn | 11466.07 | 5.34 | 30 hours | 0.507 |

**Supplementary Table 3:** Physicochemical characteristics of the target proteins


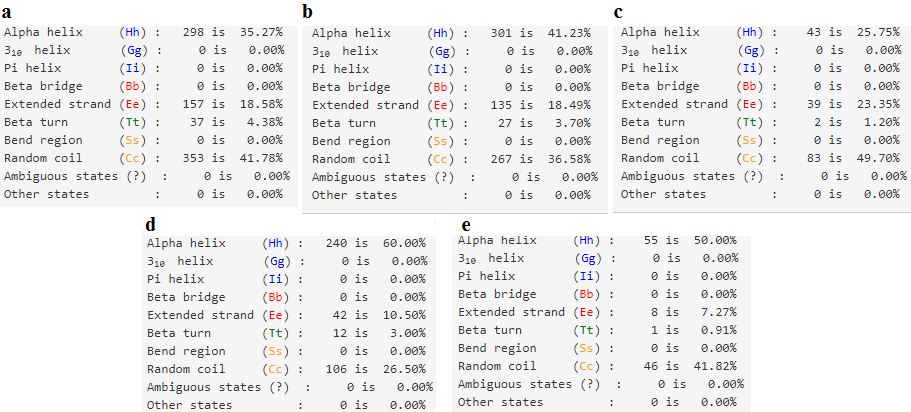


**Supplementary Figure 1:** Secondary structure properties of the target proteins of HHV 8 (A) Glycoprotein B, (B) Glycoprotein H, (C) Glycoprotein L, (D) Glycoprotein M and (E) Glycoprotein N

| **Target proteins** | **Homology modeling tools employed** | **Template** | **Ramachandran Plot analysis** |
| --- | --- | --- | --- |
| gB | I Tassser | 3fvcA | Favoured region: 69.3%  Allowed region: 16.5%  Outlier region: 14.2% |
|  | Raptor X | 3fvcA | Favoured region: 91.8%  Allowed region: 6.4%  Outlier region: 1.8% |
|  | Phyre 2 | 3fvcA | Favoured region: 88.8%  Allowed region: 4.3%  Outlier region: 6.9% |
| gH | I Tassser | 3phfA | Favoured region: 77.9%  Allowed region: 14.8%  Outlier region: 7.3% |
|  | Raptor X | 5w0kA | Favoured region: 92.4%  Allowed region: 5.8%  Outlier region: 1.8% |
|  | Phyre 2 | 3phf | Favoured region: 84%  Allowed region: 12.5%  Outlier region: 3.5% |
| gL | I Tassser | 3phfA/B | Favoured region: 58.8%  Allowed region: 26.7%  Outlier region: 14.5% |
|  | Raptor X | 5w0kB | Favoured region: 92.1%  Allowed region: 6.1%  Outlier region: 1.8% |
|  | Phyre 2 | 3phf | Favoured region: 77.1%  Allowed region: 15.6%  Outlier region: 7.3% |
| gM | I Tassser | 3d9bA | Favoured region: 81.7%  Allowed region: 13.6%  Outlier region: 4.8% |
|  | Raptor X | 1occA | Favoured region: 94.2%  Allowed region: 4.1%  Outlier region: 1.8% |
|  | Phyre 2 | 5sy1 | Favoured region: 90.7%  Allowed region: 6.8%  Outlier region: 2.5% |
| gN | I Tassser | 3mk7 | Favoured region: 53.5%  Allowed region: 28.7%  Outlier region: 17.6% |
|  | Raptor X | 5x0mA | Favoured region: 91.7%  Allowed region: 3.7%  Outlier region: 4.6% |
|  | Phyre 2 | 4jkv | Favoured region: ND  Allowed region:  Outlier region: |

**Supplementary Table 4:** The details of the homology modeling carried out for the target proteins using different tools.

| **Target protein** |  | **Template** | **p-value** | **Overall uGDT (GDT)** |
| --- | --- | --- | --- | --- |
| gB | Domain 1 | 3fvca | 2.79e-18 | 482 (57) |
|  | Domain 2 | 1cja | 2.62e-02 |  |
|  | Domain 3 | 4k1c, 4kjr, 3v5s | 8.50e-03 |  |
| gH | Domain 1 | 5W0K | 2.77e-16 | 394(54) |
| gL | Domain 1 | 5W0K | 7.44e-04 | 67 (40) |
| gM | Domain 1 | 1occ, 1m56, 1fft, 3ayf | 1.70e-04 | 97 (24) |
|  | Domain 2 | 3by7 | 7.28e-02 |  |
| gN | Domain 1 | 5x0m | 8.16e-03 | 42 (38) |

**Supplementary Table 5:** The detailed alignment statistics of the finalized models of the target proteins.

| **Protein** | **Peptide sequence (Position)** | **HLA Class II alleles** | **HLA Class I supertypes and alleles** |
| --- | --- | --- | --- |
| **gB** | VRRYRKIATSVTVYR (112) | HLA-DRB1*01:01, HLA-DRB1*04:01, HLA-DRB1*07:01, HLA-DRB1*08:03, HLA-DRB1*10:01, HLA-DRB1*11:01 | A1, A3, A26, B58, B62, HLA-B*5801 |
|  | LVTFKFLNSSNLFTG (555) | HLA-DRB1*01:01, HLA-DRB1*04:01, HLA-DRB1*07:01, HLA-DRB1*10:01, HLA-DRB1*15:01 | A24, B62, HLA-A*02:01, HLA-B*5801 |
|  | IAIILIIFMLSRRTN (743) | HLA-DRB1*11:01, HLA-DRB1*12:01, HLA-DRB1*14:01, HLA-DRB1*15:01 | A2, A3, A26, B58, B62, HLA-A*02:01, HLA-B*35:01, HLA-B*5101, HLA-B*5801 |
| **gH** | AELYFLRRISRLCMP (343) | HLA-DRB1*11:01, HLA-DRB1*12:01, HLA-DRB1*14:01, HLA-DRB1*15:01 | A24, B8, B27, B39, B44 |
|  | ADKIIATVPLPHVTY (562) | HLA-DRB1*07:01, HLA-DRB1*08:03, HLA-DRB1*10:01, HLA-DRB1*11:01, DRB1*14:01 | A1, B62, HLA-B*51:01 |
|  | IRGMYRRRAASALFL (694) | HLA-DRB1*01:01, HLA-DRB1*07:01, HLA-DRB1*10:01 | B7, B8, B27, B39, B62 |
| **gL** | GFNLRSFLVAVVRRL (77) | HLA-DRB1*07:01, HLA-DRB1*08:03, HLA-DRB1*10:01, HLA-DRB1*11:01, HLA-DRB1*12:01, DRB1*14:01, HLA-DRB1*15:01 | A2, A3, B8, B27, HLA-A*02:01, HLA-B*5101, HLA-B*5801 |
| **gM** | KSDRFLMSSWVKLLF (5) | HLA-DRB1*01:01, HLA-DRB1*07:01, DRB1*14:01, HLA-DRB1*15:01 | A1, A2, A3, A24, A39, B58,B62, HLA-A*02:01,  , HLA-B*35:01, HLA-B*5801 |
|  | VIMYICSAVVPMAAT (22) | HLA-DRB1*01:01, HLA-DRB1*04:01, HLA-DRB1*07:01, HLA-DRB1*08:03, HLA-DRB1*10:01 | A2, B8, B62, HLA-A*02:01, HLA-B*35:01 |
|  | QLFIHVLSYKHVLMA (140) | HLA-DRB1*04:01, HLA-DRB1*07:01, HLA-DRB1*10:01, HLA-DRB1*11:01, HLA-DRB1*12:01, DRB1*14:01, HLA-DRB1*15:01 | A1, A26, B62 |
|  | FCISFAHIQSLITCN (162) | HLA-DRB1*01:01, HLA-DRB1*04:01, HLA-DRB1*07:01, HLA-DRB1*10:01 | A24, B58, B62, , HLA-B*35:01 |
|  | VNLYLSTTALEMLLF (207) | HLA-DRB1*01:01, HLA-DRB1*04:01, HLA-DRB1*07:01, HLA-DRB1*10:01 | A1, A2, A26, B7, B8, B39, B58, B62, A0201 |
| **gN** | SSFSSVWALINALLV (73) | HLA-DRB1*07:01, HLA-DRB1*10:01, HLA-DRB1*15:01 | A2, A24, A26, B7, B62, HLA-B*35:01 |

**Supplementary Table 6:** Overlapping HTL and CTL epitopes

.

**
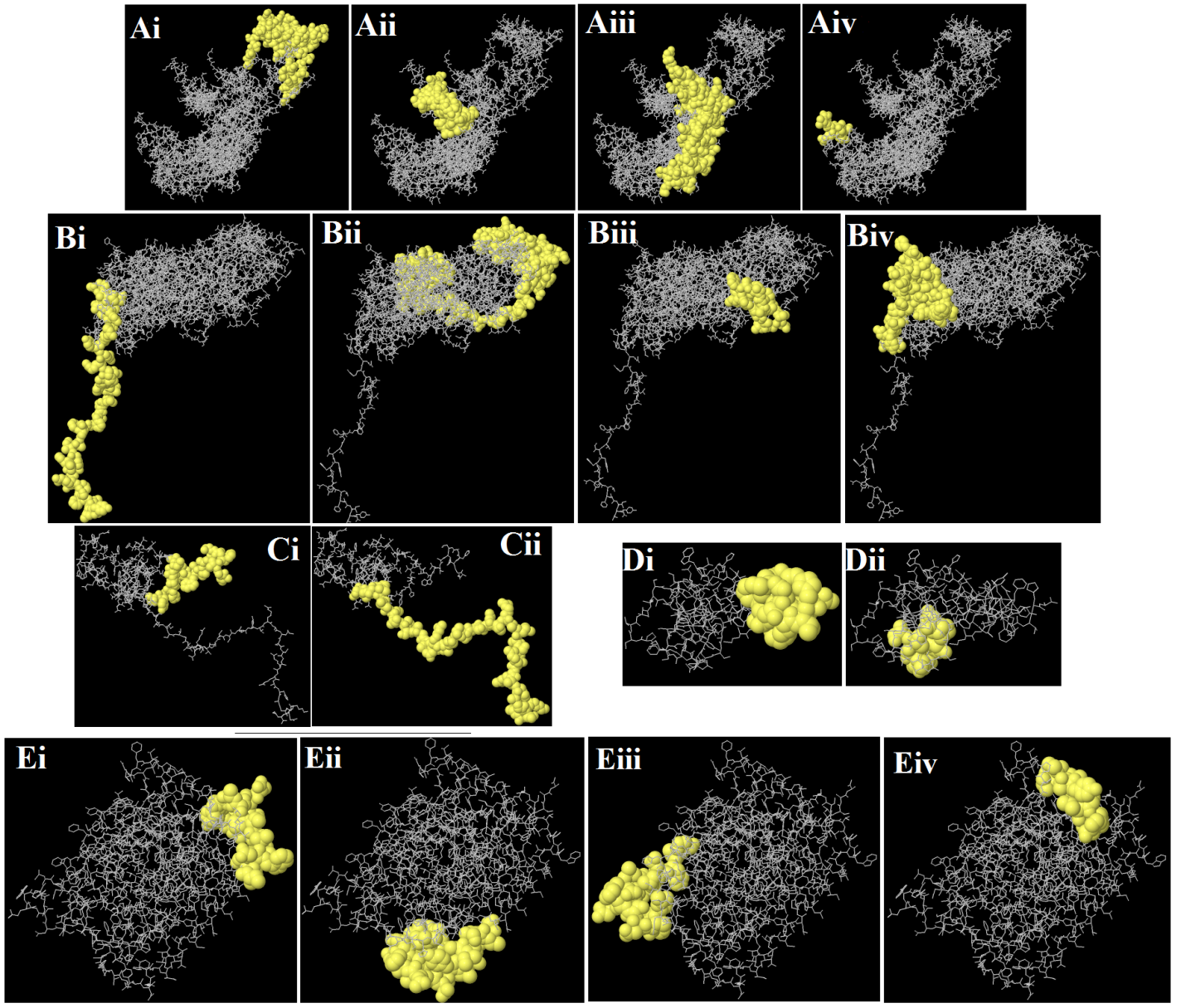
**

**Figure 3. Discontinuous/Conformational B cell epitopes.** In ball-and-stick model, the predicted epitope residues are shown as yellow balls (abbreviated amino acids) and non-epitope and core residues are shown in sticks (superscript numerals): Ai-Aiv (confirmation epitopes in gB): **Ai**- YGRPVSAKFVGDAISVTECINVDQSSVNIHKSLRTNSKDVCYARPLVTFKFLNSSNLFTGQLGARNEIILTNNQVETCKDTCEHYFITRNETLVYKDYAYLRTINTTDISTLNT^510-623^, **Aii-** GLREDLDNTIDMNKERFVRDLSEIVADLGGIGKTVVNVASSVV^673-715^, **Aiii-** LIIFMLSRRTNTIAQAPVKMIYPDVDRRAPPSGGAPTREEIKNILLGMHQLQQEERQKADDLKKSTPSVFQRTANGLRQRLRGYKPLTQSLDISPETGE^747-845^; **Aiv-** VYRGLTESAI^124-133^; Bi-Biv (confirmation epitopes in gH): **Bi-** WLRDNGTVVEIRGMYRRRAASALFLILSFIGFSGVIYFLYRLFSILY^684-730^, **Bii**: SATQLINGRTNLSIELEFNGTSFFLNWQNLLNVITEPALTELWTSAEVAEDLRVTLKKRQSLFFPNKTVVISGDGHRYTCEVPTSSQTYNITKGFNYSALPGHLGGFGINARL^36-148^, **Biii-** MQGLAFLAALACWRCISLTCGATGA^1-25^, **Biv-** PHVTYIISSEALSNAVVYEVSEIFLKSAMFISAIKPDCSGFNFSQIDRHIPIVYNISTPRRGCPLCDS^572-639^; Ci-Cii (confirmation epitopes in gL) **Ci-** MGIFALFAVLWTTLLVTSHAYV^1-22^, **Cii-** DSSIHNVNIIIISVGKAMNRTGSVSGSQTRAKSSSRRAHAGQKGK^123-167^, Di-Dii (confirmation epitopes in gN): **Di-** YLVYLCFFKFVDEVVHA^94-110^; **Dii-** SVTTPGFYDV^52-61^, Ei-Eiv (confirmation epitopes in gM): **Ei**- IRSDWGLCTPSAAYMPLATSA^324-344^, **Eii-** LPVIRYENAFVQANLHYIVAINISCI^286-311^, **Eiii**- LITCNSAQWEIPLLEQHVP^172-190^, **Eiv-** YYCLAAVAVYRAKHVHATTMMSMQSW^94-119^

| **Glycoproteins** | **Position of the epitopes** | **Epitope sequence** |
| --- | --- | --- |
| gB | 121-136 | VTVYRGLTESAITNK |
|  | 122-137 | TVYRGLTESAITNKY |
|  | 123-138 | VYRGLTESAITNKYE |
|  | 124-139 | YRGLTESAITNKYEL |
|  | 125-140 | RGLTESAITNKYELP |
|  | 126-141 | GLTESAITNKYELPR |
|  | 127-142 | LTESAITNKYELPRP |
|  | 128-143 | TESAITNKYELPRPV |
|  | 162-177 | VNVNGVENTFTDRDD |
|  | 163-178 | NVNGVENTFTDRDDV |
|  | 208-223 | WFPGIYRVRTTVNCE |
|  | 209-224 | FPGIYRVRTTVNCEI |
|  | 210-225 | PGIYRVRTTVNCEIV |
|  | 211-226 | GIYRVRTTVNCEIVD |
|  | 212-227 | IYRVRTTVNCEIVDM |
|  | 213-228 | YRVRTTVNCEIVDMI |
|  | 214-229 | RVRTTVNCEIVDMIA |
|  | 215-230 | VRTTVNCEIVDMIAR |
|  | 229-244 | RSAEPYNYFVTSLGD |
|  | 230-245 | SAEPYNYFVTSLGDT |
|  | 231-246 | AEPYNYFVTSLGDTV |
|  | 232-247 | EPYNYFVTSLGDTVE |
|  | 233-248 | PYNYFVTSLGDTVEV |
|  | 234-249 | YNYFVTSLGDTVEVS |
|  | 235-250 | NYFVTSLGDTVEVSP |
|  | 236-251 | YFVTSLGDTVEVSPF |
|  | 327-342 | QTTHEDSFHFVANEI |
|  | 328-343 | TTHEDSFHFVANEIT |
|  | 336-351 | FVANEITATFTAPLT |
|  | 386-401 | TVQYFHTTGGLYLVW |
|  | 639-653 | AIELYSSAEKRLASS |
|  | 640-654 | IELYSSAEKRLASSV |
|  | 641-655 | ELYSSAEKRLASSVF |
|  | 658-672 | ETMFREYNYYTHRLA |
|  | 659-673 | TMFREYNYYTHRLAG |
|  | 660-674 | MFREYNYYTHRLAGL |
|  | 661-675 | FREYNYYTHRLAGLR |
|  | 662-676 | REYNYYTHRLAGLRE |
|  | 663-677 | EYNYYTHRLAGLRED |
|  | 664-678 | YNYYTHRLAGLREDL |
|  | 708-722 | VNVASSVVTLCGSLV |
|  | 709-724 | NVASSVVTLCGSLVT |
|  | 710-725 | VASSVVTLCGSLVTG |
|  | 711-726 | ASSVVTLCGSLVTGF |
|  | 712-727 | SSVVTLCGSLVTGFI |
|  | 713-728 | SVVTLCGSLVTGFIN |
|  | 714-729 | VVTLCGSLVTGFINF |
|  | 803-817 | QKADDMKKSTPSVFQ |
|  | 804-818 | KADDMKKSTPSVFQR |
|  | 805-819 | ADDMKKSTPSVFQRT |
|  | 806-820 | DDMKKSTPSVFQRTA |
|  | 807-821 | DMKKSTPSVFQRTAN |
|  | 808-822 | MKKSTPSVFQRTANG |
|  | 809-823 | KKSTPSVFQRTANGL |
|  | 810-824 | KSTPSVFQRTANGLR |
|  | 811-825 | STPSVFQRTANGLRQ |
| gH | 78-93 | TSAEVAEDLRVTLKK |
|  | 79-94 | SAEVAEDLRVTLKKR |
|  | 80-95 | AEVAEDLRVTLKKRQ |
|  | 81-96 | EVAEDLRVTLKKRQS |
|  | 82-97 | VAEDLRVTLKKRQSL |
|  | 83-98 | AEDLRVTLKKRQSLF |
|  | 84-99 | EDLRVTLKKRQSLFF |
|  | 152-167 | IFASKWSLFARDTPE |
|  | 153-168 | FASKWSLFARDTPEY |
|  | 154-169 | ASKWSLFARDTPEYR |
|  | 155-170 | SKWSLFARDTPEYRV |
|  | 156-171 | KWSLFARDTPEYRVF |
|  | 157-172 | WSLFARDTPEYRVFY |
|  | 225-240 | PDSLPSLKGHATYDE |
|  | 226-241 | DSLPSLKGHATYDEL |
|  | 227-242 | SLPSLKGHATYDELT |
|  | 228-243 | LPSLKGHATYDELTF |
|  | 229-244 | PSLKGHATYDELTFA |
|  | 230-245 | SLKGHATYDELTFAR |
|  | 234-249 | HATYDELTFARNAKY |
|  | 235-250 | ATYDELTFARNAKYA |
|  | 236-251 | TYDELTFARNAKYAL |
|  | 237-252 | YDELTFARNAKYALV |
|  | 238-253 | DELTFARNAKYALVA |
|  | 268-283 | TRIFLNMTESTPLEF |
|  | 269-284 | RIFLNMTESTPLEFT |
|  | 270-285 | IFLNMTESTPLEFTR |
|  | 271-286 | FLNMTESTPLEFTRT |
|  | 272-287 | LNMTESTPLEFTRTI |
|  | 273-288 | NMTESTPLEFTRTIQ |
|  | 275-290 | TESTPLEFTRTIQTR |
|  | 276-291 | ESTPLEFTRTIQTRI |
|  | 277-292 | STPLEFTRTIQTRIV |
|  | 338-353 | CRQYAELYFLRRISR |
|  | 339-354 | RQYAELYFLRRISRL |
|  | 340-355 | QYAELYFLRRISRLC |
|  | 578-592 | ISSEALSNAVVYEVS |
|  | 579-593 | SSEALSNAVVYEVSE |
|  | 580-594 | SEALSNAVVYEVSEI |
| gL | 58-73 | IAKLRSKTGDITVET |
|  | 59-74 | AKLRSKTGDITVETC |
|  | 78-93 | NLRSFLVAVVRRLGS |
|  | 79-94 | LRSFLVAVVRRLGSW |
|  | 80-95 | RSFLVAVVRRLGSWA |
|  | 81-96 | SFLVAVVRRLGSWAS |
|  | 82-97 | FLVAVVRRLGSWASQ |
|  | 83-98 | LVAVVRRLGSWASQE |
|  | 95-110 | SQENLRLLWYLQRSL |
|  | 96-111 | QENLRLLWYLQRSLT |
|  | 97-112 | ENLRLLWYLQRSLTA |
|  | 98-113 | NLRLLWYLQRSLTAY |
|  | 99-114 | LRLLWYLQRSLTAYT |
|  | 100-115 | RLLWYLQRSLTAYTV |
|  | 101-116 | LLWYLQRSLTAYTVG |
|  | 102-117 | LWYLQRSLTAYTVGF |
| gM | 21-36 | VIMYICSAVVPMAAT |
|  | 170-185 | SLITCNSAQWEIPLL |
|  | 171-186 | LITCNSAQWEIPLLE |
|  | 172-187 | ITCNSAQWEIPLLEQ |
|  | 173-188 | TCNSAQWEIPLLEQH |
|  | 174-189 | CNSAQWEIPLLEQHV |
|  | 260-275 | FFLVKFMRRQVGFYV |
|  | 261-276 | FLVKFMRRQVGFYVG |
|  | 262-277 | LVKFMRRQVGFYVGV |
|  | 263-278 | VKFMRRQVGFYVGVF |
|  | 264-279 | KFMRRQVGFYVGVFV |
|  | 265-280 | FMRRQVGFYVGVFVG |
|  | 266-281 | MRRQVGFYVGVFVGY |
|  | 267-282 | RRQVGFYVGVFVGYL |
|  | 372-387 | ISTPAPRTQYQSDHE |
| gN | 84-99 | LLVVVATFFYLVYLC |
|  | 85-100 | LVVVATFFYLVYLCF |
|  | 86-101 | VVVATFFYLVYLCFF |
|  | 87-102 | VVATFFYLVYLCFFK |

**Supplementary Table 7:** IFN-γ inducing epitopes predicted by IFN epitope server.

|  | **Epitopes** | **Population Coverage (Worldwide) %** |
| --- | --- | --- |
| **CTL Epitopes** | Gb-I-150 | 58.3 |
|  | Gb-I-363 | 62.36 |
|  | Gh-I-340 | 66.58 |
|  | Gh-I-417 | 64.80 |
|  | Gl-I-13 | 68.60 |
|  | Gm-I-210 | 66.30 |
|  | Gm-I-249 | 67.17 |
| **HTL Epitopes** | Gb-II-555 | 72.40 |
|  | Gb-II-743 | 77.53 |
|  | Gh-II-694 | 91.30 |
|  | Gl-II-77 | 85.96 |
|  | Gm-II-140 | 92.57 |
|  | Gm-II-162 | 71.34 |
|  | Gm-II-207 | 89.08 |

**Supplementary Table 8:** Worldwide population coverage (in %) of the predicted CTL and HTL epitopes predicted by IEDB population coverage analysis


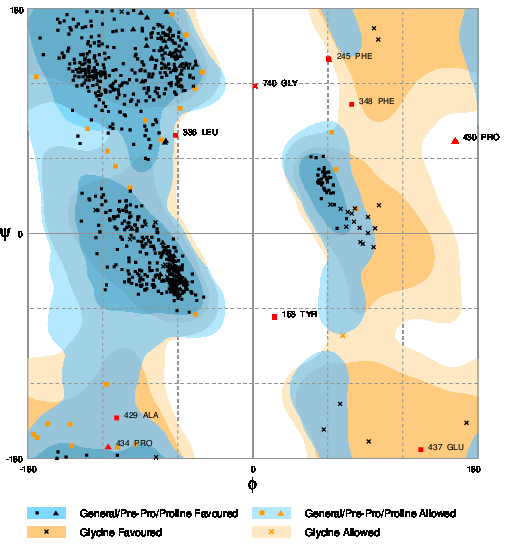


**Supplementary Figure 3:** Ramachandran Plot analysis of the refined model of TLR-9. 95.6%, 3.2% and 1.2% regions are in the favoured, allowed and outlier regions.


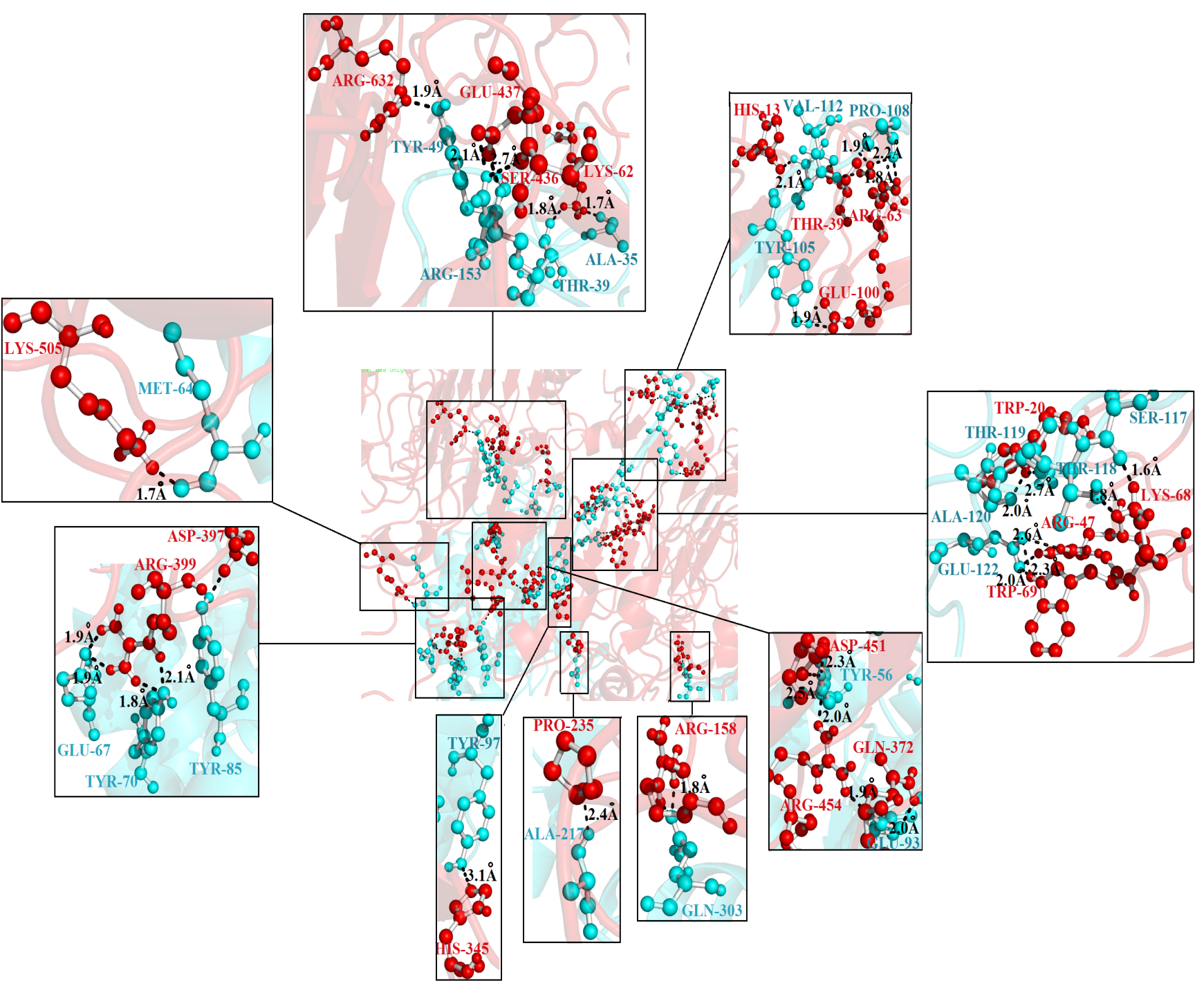


**Supplementary Figure 4:** The detailed overview of the interaction pattern between TLR-9 and the final vaccine construct. The amino acids depicted in red and cyan colour belongs to TLR-9 and the vaccine respectively.
